# Frequency and correlates of non-receipt of age-appropriate vaccination among children aged 6-35 months with medically attended diarrhea: Findings from the Enterics for Global Health (EFGH) *Shigella* study, 2022-2024

**DOI:** 10.1371/journal.pgph.0005670

**Published:** 2026-07-01

**Authors:** Caren Oreso, Billy Ogwel, Alex O. Awuor, Raphael O. Anyango, Karen Kotloff, M. Jahangir Hossain, Henry Badji, Khuzwayo C. Jere, Latif Ndeketa, Josh Colston, Katia Manzanares Villanueva, Firdausi Qadri, Md. Taufiqul Islam, Farah Naz Qamar, Naveed Ahmed, Sonia I. Rao, Patricia B. Pavlinac, Richard Omore

**Affiliations:** 1 Kenya Medical Research Institute-Center for Global Health Research (KEMRI-CGHR), Kisumu, Kenya; 2 Centre pour le Développement des Vaccins du Mali (CVD-Mali), Bamako, Mali; 3 Center for Vaccine Development and Global Health, University of Maryland School of Medicine, Baltimore, Maryland, United States of America; 4 Department of Medicine, University of Maryland School of Medicine, Baltimore, Maryland, United States of America; 5 Medical Research Council Unit The Gambia at the London School of Hygiene and Tropical Medicine, Banjul, The Gambia; 6 Malawi Liverpool Wellcome Programme, Blantyre, Malawi; 7 Department of Medical Laboratory Sciences, School of Life Sciences and Health Professions, Kamuzu University of Health Sciences, Blantyre, Malawi; 8 Institute of Infection, Veterinary and Ecological Sciences, University of Liverpool, Liverpool, United Kingdom; 9 Department of Genetics, University of Cambridge, Cambridge, United Kingdom; 10 Department of Environmental and Community Health, School of Global Public Health, Kamuzu University of Health Sciences, Blantyre, Malawi; 11 Division of Infectious Diseases and International Health, The University of Virginia School of Medicine, Charlottesville, Virginia, United States of America; 12 Asociación Benéfica Prisma, Unidad de Investigaciones Biomedicas, Iquitos, Loreto, Peru; 13 International Centre for Diarrhoeal Disease Research, Bangladesh, Dhaka, Bangladesh (icddr, b), Dhaka, Bangladesh; 14 Department of Pediatrics and Child Health, The Aga Khan University, Karachi, Pakistan; 15 Department of Global Health, University of Washington, Seattle, Washington, United States of America; Christian Medical College Vellore, INDIA

## Abstract

Complete childhood immunization protects children from long-term health complications and disabilities caused by vaccine-preventable diseases. Enterics for Global Health (EFGH)-*Shigella* surveillance was a two-year study measuring incidence rates and consequences of *Shigella* among children aged 6–35 months in seven sites located in Asia, Latin America and Africa. Here, we estimated the prevalence and factors associated with non-receipt of age-appropriate vaccination among children enrolled in the EFGH-*Shigella* study. In this nested cross-sectional study, we analysed data from 7,932 children aged 6–35 months presenting with medically attended diarrhea (MAD). Vaccines recommended per each country’s national immunization schedule were extracted from medical records and risk factors were collected by caregiver interview and physical exam. We defined age-appropriate vaccines as receipt of the early childhood vaccinations within one month of the recommended age, according to the national immunization schedule, on the immunization card. Poison regression was used to identify independent factors associated with non-receipt of age-appropriate vaccination accounting for all covariates. Over half of enrolled children (51.7%) did not receive all age-appropriate vaccines most commonly in The Gambia (75.2%) and least frequently in Bangladesh (22.3%). Children 12–35 months of age were more likely not have all age appropriate vaccines compared to children 6–11 months (aPR: 1.47, 95%CI 1.39 to 1.54), children who came from households with ≥3 children aged <5 years (aPR:1.07;1.01-1.13), had mothers with low education (aPR:1.18; 1.12-1.24), and were wasted (Moderately: aPR: 1.06; 1.00-1.13; Severely: aPR: 1.13, 1.03-1.24) were more likely to miss all age-appropriate vaccines compared to their counterparts who did not. Non-receipt of age-appropriate vaccination was largely age dependent, driven by mother’s education and severe wasting highlighting the need to design effective strategies that incorporate site complexities to improve timely vaccination targeting vulnerable groups.

## Introduction

Infectious diseases are the major cause of morbidity and mortality in low-and-middle-income countries (LMICs) [1]. Globally, in 2019, the proportion of disability-adjusted life-years (DALYs) associated with potential vaccine preventable infectious diseases in children under-5 years was highest in sub-Saharan Africa, South Asia and Latin America [2]; the same places with the lowest vaccine uptake [3]. In 2019, vaccine preventable deaths from diarrhea alone accounted for nearly ten percent of all under-5 deaths [1].

Immunization is a proven lifesaving intervention and several vaccines exist for infectious diseases such as rotavirus, measles, tuberculosis, tetanus, diphtheria, polio, and pertussis. However, despite the preventability of these diseases and universal availability of early childhood vaccinations, these conditions continue to be the main drivers of morbidity and mortality in LMICs [2]. In 2023, two years after the World Health Organization (WHO) kicked off the ambitious new Immunization Agenda 2030 (IA2030), 40.8% of children did not receive any diphtheria, pertussis, and tetanus vaccinations [4]. Few studies have quantitatively examined age-appropriate vaccination in LMICs and those that have, are often limited by small sample sizes and focused on single-country contexts [5–7]. By leveraging data from the Enterics for Global Health (EFGH) *Shigella* surveillance study—which spans countries across Africa, South Asia, and Latin America—our study provides an added advantage of a large, diverse sample that provides broader insights across multiple settings with demand for vaccination against infectious diseases. Understanding factors of non-receipt of age appropriate vaccination in LMICs could help inform resource allocation and strategies aimed at optimising vaccine uptake, and help achieve child health targets.

EFGH study is a two-year study designed to establish baseline incidence rates and consequences of *Shigella* diarrhea with the intent to inform *Shigella* vaccine trials and introduction in countries with the highest disease burden [8]. Here, we estimated prevalence and factors associated with non-receipt of age-appropriate vaccination among children aged 6–35 months enrolled with medically attended diarrhea (MAD) in the EFGH study.

## Methods

### Study setting and population

Between June 2022 and August 2024, The EFGH Study was conducted using a prospective, hybrid surveillance study across seven countries: Bangladesh, Kenya, Malawi, Mali, Pakistan, Peru and The Gambia. Over this 24-month period, each country team recruited a maximum of 1,400 children presenting with acute diarrhea at study health facilities. Eligibility was defined as the onset of a new diarrheal episode within the last week (preceded by at least 2 diarrhea-free days) in a child living within the specified catchment area. A diarrheal episode was clinically defined as the passage of three or more loose or watery stools, with or without blood, in the 24 hours prior to presentation. All participants were required to be accompanied by a caregiver who provided informed consent. Detailed methods [8] along with characteristics of the study settings and populations are described in detail elsewhere [9–15].

### Definitions

*Vaccine completeness* included assessing vaccine receipt and timing of the following vaccines*:* Bacillus Calmette-Guérin (BCG), Polio, DPT (Diphtheria, Pertussis and Tetanus) Hepatitis B, *Haemophilus influenzae* type b (Hib), Rotavirus, Measles-containing vaccine (MCV) (Measles+Mumps+Rubella; Measles+Rubella; Measles) and Pneumococcal conjugate vaccine (PCV). Rotavirus vaccination has not been introduced into the national immunization schedule in Bangladesh therefore considered vaccines receipt as above except Rotavirus to meet the complete vaccine definition in Bangladesh. The country-specific routine immunization schedules are shown in S1 Table.

*Non-receipt of age-appropriate vaccination* was defined as absence of a record of having received all the eight (or seven for Bangladesh) vaccinations within one month of the recommended age, according to the national immunization schedule, on the immunization card. For each individual vaccine, non-receipt was defined as the absence of card-confirmed documentation of the age-appropriate dose(s) in accordance with the national immunization schedule, allowing for a one-month delay.

*Diarrhea* was defined as a caregiver report of three or more abnormally loose or watery stools in the previous 24 hours.

*Medically attended diarrhea (MAD)* was defined as diarrhea that led to seeking care at a designated EFGH study sentinel facility by the caregiver.

Household socioeconomic status was measured using asset-based wealth indices derived from country-specific indicators, including housing characteristics, access to water and sanitation, and ownership of durable assets. A composite score was generated as a weighted sum of these variables, following established Demographic and Health Surveys (DHS) methodology [16]. Two indices were constructed: a national wealth index, which used country-specific weights and cut-offs to assign households into national quintiles, and where applicable (Bangladesh, Malawi, Mali, Pakistan, and Peru), an urban-specific index, derived using weights and quintiles restricted to urban populations to better capture within-urban socioeconomic variation. Depending on whether the study catchment area was urban or rural/mixed, participants were assigned wealth quintiles using the corresponding index. Quintiles were categorized as 1 (lowest) to 5 (highest) wealth.

### Study design

This was a secondary, cross-sectional analysis of baseline data collected in the EFGH study.

*Inclusion criteria*: Children with MAD aged 6–35 months, residing in a demographically defined study catchment population, who sought care at a designated study sentinel health facility with a vaccination card, planned to remain at their current residence for at least 4 months, and whose primary caregiver was able to provide written informed consent, were included in this analysis.

*Exclusion criteria:* Children were excluded if younger than 6 months or older than 35 months and presented with non-diarrhea conditions, if the caregiver-reported diarrhea did not meet the study definition, if the primary caregiver did not provide written informed consent or did not reside in the study catchment area, if vaccination cards were not available, if four or more hours passed between when the child presented at the health facility and the study screening occurred, if the site enrolment sample size had been met, or if child was referred to a non-study facility for further treatment.

### Data collection

The information collected included basic patient and family demographics, geographic, socio-economic and clinical characteristics, water and sanitation variables as described elsewhere [17]. Vaccination information was abstracted from the immunization cards.

### Statistical analysis

Frequencies and percentages were used to summarize the vaccination status of children with reported MAD. Overall and site-specific estimates for each vaccine and overall age-appropriate vaccination were calculated. We estimated both overall and site-specific prevalence of zero-dose status and full immunization among children aged ≥12 months. Zero-dose status was defined using two complementary criteria: (i) children who had not received the first dose of a diphtheria–tetanus–pertussis (DTP)-containing vaccine; and (ii) children who had not received any doses of Bacille Calmette–Guérin (BCG), polio, pentavalent, or measles-containing vaccines (MCV) [18]. Full immunization was defined as receipt of all eight doses recommended under the Expanded Programme on Immunization (EPI): one dose of BCG, three doses each of pentavalent and polio vaccines, and one dose of a measles-containing vaccine [19]. We employed descriptive statistics to summarize factors associated with non-receipt of age-appropriate vaccination. Continuous variables were presented using median and interquartile range, and comparisons between groups were performed using the Wilcoxon Rank-Sum test. Categorical variables were summarized using frequencies and proportions, and group comparisons were conducted using the Chi-square test.

Poisson regression was used to identify factors associated with non-receipt of age-appropriate vaccination; the main binary outcome of interest which has been described previously [20]. Initially, all potential predictor factors were assessed in separate individual bivariate Poisson regression models. Variables with a p-value < 0.2 were included and retained in the final multivariable in the model. To estimate the crude and adjusted prevalence ratios crude and adjusted and their corresponding 95% confidence intervals, we employed Poisson regression with robust standard errors to account for potential overdispersion and clustering at the country level. The goodness-of-fit of the final model was assessed using the deviance-to-degrees-of-freedom ratio, with values approaching 1.0 indicating adequate model fit.

We also conducted sensitivity analyses, stratifying the models by age and site, separately.

### Ethical considerations

This analysis involved data collected as part of EFGH protocol which was approved by the respective IRBs at all the country sites prior to study initiation [8]. All parents/legally authorized representatives (LAR) provided written informed consent [8].

## Results

**Fig 1:**
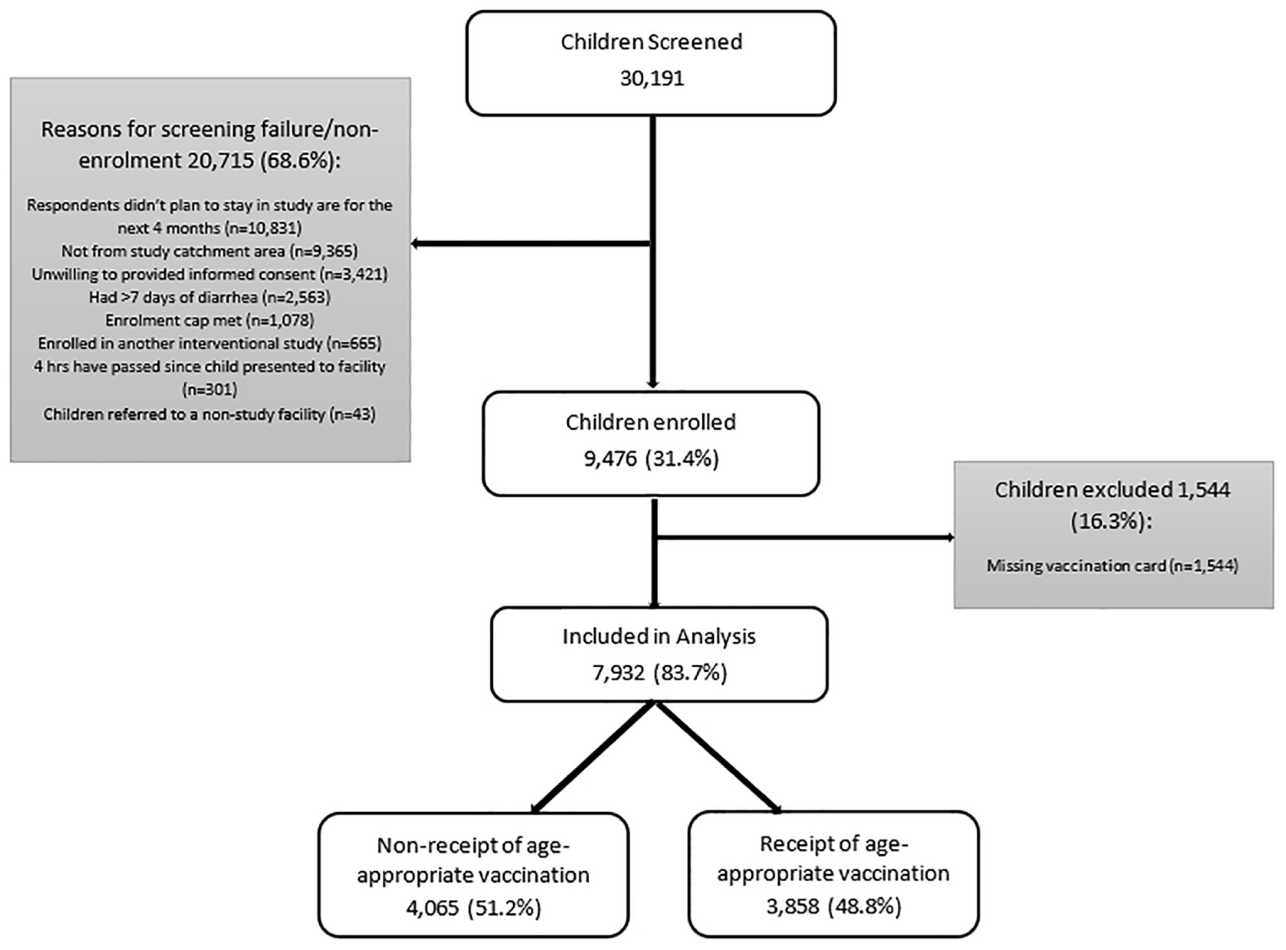
Flowchart of enrolment of children aged 6-35 months presenting with medically attended diarrhea and vaccination status, EFGH *Shigella* surveillance study, June 2022 - August 2024.

From June 2022 to August 2024, we screened 30,191 children with MAD; of whom 9,476 (31.4%) were enrolled in the EFGH study (Fig 1).

We excluded 1,544 (16.3%) children in this secondary analysis due to missing a vaccine card. Of the 7,932 children included, the median (interquartile range [IQR]) age of the children analysed was 14 (9–21) months and 4,296 (54.2%) children were male. The caregiver’s median (IQR) age was 25 (22–30) years and 52.2% of these were mothers who had low education (primary school only or no education). Approximately 22% of children were stunted. Majority (76.0%) of caregivers were not working at the time the child was enrolled in this study. Among the children enrolled 4,617 (58.2%), 1,385 (17.5%), 1,930 (24.3%) had mild, moderate and severe diarrhea, respectively (Table 1).

**Table 1:** Characteristics of study population stratified by vaccination status, EFGH *Shigella* surveillance study, June 2022 - August 2024.

Variable	Category	Overall	Non-receipt of Age appropriate Vaccination (N = 4,100, 51.7%)	Age-appropriate Vaccination (N = 3,832, 48.3%)
Demographic characteristics		n (%)	n (%)	n (%)
Age in months	Median [Q1–Q3]	14 [9–21]	16 [10–22]	12 [8–16]
Age category	6-11m	3167 (39.9)	1264 (30.8)	1903 (49.7)
	12-17m	2145 (27.0)	1026 (25.0)	1119 (29.2)
	18-23m	1454 (18.3)s	935 (22.8)	519 (13.5)
	24-35m	1166 (14.7)	875 (21.3)	291 (7.6)
sex	Male	4296 (54.2)	2189 (53.4)	2107 (55)
Site	Bangladesh	1211 (15.3)	270 (6.6)	941 (24.6)
	Kenya	1211 (15.3)	867 (21.1)	344 (9.0)
	Malawi	954 (12.0)	542 (13.2)	412 (10.8)
	Mali	1229 (15.5)	506 (12.3)	723 (18.9)
	Pakistan	992 (12.5)	527 (12.9)	465 (12.1)
	Peru	946 (11.9)	344 (8.4)	602 (15.7)
	The Gambia	1389 (17.5)	1044 (25.5)	345 (9.0)
No. of children <5 yrs in household	**<3**	5663 (71.4)	2718 (66.3)	2945 (76.9)
	≥3	2269 (28.6)	1382 (33.7)	887 (23.1)
**Socio-economic characteristics**				
Caregiver age in years	Median [Q1–Q3]	25 [22–30]	26 [22–30]	25 [22–30]
	<20 yrs	731 (9.2)	357 (8.7)	374 (9.8)
	20–24 yrs	2564 (32.3)	1313 (32.0)	1251 (32.6)
	25–29 yrs	2188 (27.6)	1141 (27.8)	1047 (27.3)
	30–34 yrs	1409 (17.8)	745 (18.2)	664 (17.3)
	≥35 yrs	1040 (13.1)	544 (13.3)	496 (12.9)
Final Quintile	Quintile 1 - Least wealthy	1238 (15.6)	717 (17.5)	521 (13.6)
	Quintile 2	2252 (28.4)	1256 (30.6)	996 (26.0)
	Quintile 3	2158 (27.2)	1120 (27.3)	1038 (27.1)
	Quintile 4	1498 (18.9)	755 (18.4)	743 (19.4)
	Quintile 5 - Wealthiest	786 (9.9)	252 (6.1)	534 (13.9)
Mother’s education^a^	High Education	3782 (47.8)	1650 (40.3)	2132 (55.7)
	Low Education	4137 (52.2)	2442 (59.7)	1695 (44.3)
Father’s education^a^	High Education	4190 (56.0)	1951 (51.0)	2239 (61.2)
	Low Education	3293 (44.0)	1874 (49.0)	1419 (38.8)
Caregiver occupation^b^	No employment	5977 (76.0)	2855 (70.1)	3122 (82.2)
	Formal employment	143 (1.8)	57 (1.4)	86 (2.3)
	Informal employment	1747 (22.2)	1158 (28.5)	589 (15.5)
**Clinical characteristics**				
Diarrhea duration in days	Median [Q1–Q3]	4 [3–6]	4 [3–6]	4 [3–6]
Respiratory rate^c^	Normal	6980 (88.0)	3632 (88.6)	3348 (87.4)
	Low	562 (7.1)	208 (5.1)	354 (9.2)
	High	390 (4.9)	260 (6.3)	130 (3.4)
Heart rate^c^	Normal	7198 (91.7)	3775 (93.8)	3423 (89.5)
	Low	644 (8.2)	243 (6.0)	401 (10.5)
	High	7 (0.1)	6 (0.1)	1 (0.0)
Dysentery	Yes	1033 (13.0)	576 (14.0)	457 (11.9)
Prolonged^d^	Yes	1518 (19.1)	646 (15.8)	872 (22.8)
Persistent^e^	Yes	174 (2.2)	69 (1.7)	105 (2.7)
Dehydration	No sign of Dehydration	5957 (75.1)	3061 (74.7)	2896 (75.6)
	Severe	97 (1.2)	74 (1.8)	23 (0.6)
	Some	1878 (23.7)	965 (23.5)	913 (23.8)
Any subsequent episodes	Yes	2680 (33.8)	1334 (32.5)	1346 (35.1)
Modified Vesikari Score	Mild	4617 (58.2)	2436 (59.4)	2181 (56.9)
	Moderate	1385 (17.5)	715 (17.4)	670 (17.5)
	Severe	1930 (24.3)	949 (23.1)	981 (25.6)
Stunted	No	6167 (78.2)	3094 (75.8)	3073 (80.7)
	Yes	1724 (21.8)	989 (24.2)	735 (19.3)
Wasted	None	6545 (82.9)	3318 (81.2)	3227 (84.7)
	Moderate	1061 (13.4)	585 (14.3)	476 (12.5)
	Severe	290 (3.7)	181 (4.4)	109 (2.9)
Prior care-seeking	Yes	1707 (21.5)	646 (15.8)	1061 (27.7)
Seeking care in the future	Easy	7156 (90.8)	3641 (89.3)	3515 (92.5)
	Challenging	724 (9.2)	437 (10.7)	287 (7.5)
Accept new vaccine	Never	83 (1.1)	43 (1.1)	40 (1.1)
	Sometimes	440 (5.7)	275 (6.9)	165 (4.4)
	Yes	7216 (93.2)	3680 (92.0)	3536 (94.5)
Drinking water	Improved	7423 (93.6)	3761 (91.7)	3662 (95.6)
	Unimproved	509 (6.4)	339 (8.3)	170 (4.4)
Sanitation	Improved	6517 (82.2)	3215 (78.4)	3302 (86.2)
	Unimproved	1415 (17.8)	885 (21.6)	530 (13.8)
Breastfeeding	Yes	476 (6)	264 (6.5)	212 (5.6)

^a^ High education- some secondary, and secondary school or greater; Low education: No education, less than primary, primary school only, and Koranic school only.

^b^ No employment-Not employed, housewife, and student; Formal employment- professional; Informal employment-business (self-employed), business (other employer), casual laborer, and farmer.

^c^ Cutoffs for heart rate and respiratory rate based on the Pediatric Advanced Life Support (PALS) guidelines.

^d^  ≥ 7 days of diarrhea during index diarrhea episode

^e^  ≥ 14 days of diarrhea during index diarrhea episode

### Prevalence of non-receipt of age-appropriate vaccination

Among the 7,932 children included, 4,100 (51.7%) had not received all age-appropriate vaccinations at the time of enrolment (Fig 1). In general, the prevalence of non-receipt of age-appropriate vaccination varied across the study sites with The Gambia reporting the highest (75.2%) and Bangladesh the lowest prevalence (22.3%) (Table 2). Overall, the three vaccines with the highest prevalence of age-appropriate non-receipt were polio, MCV and PCV at 26.8%,19.3% and 14.1%, respectively. The vaccine with the lowest non-receipt of age appropriate vaccination uptake across all sites was BCG (2.5%).

**Table 2:** Proportion of children 6-35 months presenting with medically-attended diarrhea who had non-receipt of age-appropriate vaccination, by vaccine and age stratified by sites, EFGH *Shigella* surveillance study, June 2022 - August 2024.

	Bangladesh % [95% CI]	Kenya % [95% CI]	Malawi % [95% CI]	Mali % [95% CI]	Pakistan % [95% CI]	Peru % [95% CI]	The Gambia % [95% CI]	Overall % [95% CI]
**Vaccination**								
Any vaccination incomplete	22.3[20.0-24.8]	71.6[68.9-74.1]	56.8[53.6-60.0]	41.2[38.4-44.0]	53.1[50.0-56.3]	36.4[33.3-39.5]	75.2[72.8-77.4]	51.7[50.6-52.8]
BCG	0.2 [0.1-0.8]	1.6[1.0-2.5]	0.8[0.4-1.7]	2.4[1.7-3.5]	3.5[2.5-4.9]	3.0[2.0-4.3]	5.3[4.2-6.6]	2.5[2.1-2.8]
Polio	9.8[8.2-11.7]	39.6[36.9-42.5]	45.9[42.7-49.1]	36.6[33.9-39.4]	37.8[34.8-40.9]	18.5[16.1-21.2]	6.1[5.0-7.6]	26.8[25.8-27.8]
DPT	8.5[7.0-10.3]	1.1[0.6-1.9]	4.2[3.0-5.7]	4.6[3.6-6.0]	20.2[17.7-22.8]	18.7[16.3-21.4]	32.7[30.2-35.2]	13.2[12.4-13.9]
HEPB	8.5[7.0-10.3]	1.1[0.6-1.9]	17.8[15.5-20.4]	4.6[3.6-6.0]	38.7[35.7-41.8]	3.8[2.7-5.3]	7.2[5.9-8.7]	10.9[10.2-11.6]
HIB	8.5[7.0-10.3]	1.1[0.6-1.9]	17.8[15.5-20.4]	4.6[3.6-6.0]	20.2[17.7-22.8]	5.2[3.9-6.8]	5.0[3.9-6.3]	8.3[7.7-9.0]
Rotavirus	–	32.8[30.2-35.5]	3.7[2.6-5.1]	5.0[3.8-6.4]	14.7[12.6-17.1]	9.3[7.6-11.4]	1.7[1.1-2.5]	11.2[10.4-11.9]
MCV	15.5[13.6-17.7]	24.4[22.1-27]	14.4[12.2-16.8]	12.9[11.1-15]	16.8[14.6-19.3]	10.3[8.4-12.4]	34.8[32.3-37.4]	19.3[18.4-20.2]
PCV	8.8[7.3-10.6]	1.9[1.2-2.9]	7.8[6.2-9.7]	5.7[4.5-7.2]	20.3[17.8-22.9]	6.7[5.2-8.5]	41.9[39.3-44.6]	14.1[13.4-14.9]
**Age Category**								
6-8m	21.2[16.6-26.6]	67.7[62.3-72.7]	49.3[42.4-56.2]	27.9[23.0-33.3]	50.0 [43.0-57.0]	14.5[9.8-20.9]	44.7[37.9-51.7]	40.3[38.0-42.7]
9-11m	14.9[10.8-20.1]	62.3[55.5-68.6]	52.2[45.2-59.1]	32.9[27.2-39.2]	42.7[34.9-50.8]	16.1[11.0-23.0]	53.2[46.6-59.6]	39.4[36.9-42.0]
12-17m	21.2[16.9-26.2]	60.6[55.0-65.9]	58.4[52.0-64.5]	43.3[37.8-48.9]	48.0 [41.9-54.1]	23.7[19.0-29.1]	74.7[70.0-79.0]	47.8[45.7-50.0]
18-35m	29[24.5-33.9]	91.4[87.8-94.0]	64.4[58.5-69.9]	56.5[51.1-61.7]	62.9[57.7-67.8]	69.6[64.2-74.5]	95.8[93.7-97.2]	69.1[67.3-70.8]

BCG: Bacillus Calmette-Guérin, DPT: Diphtheria-Pertussis-Tetanus, HEPB: Hepatitis B, Hib: *Haemophilus influenzae* type b, MCV: Measles-containing vaccine and PCV: Pneumococcal conjugate vaccine.

The overall prevalence of zero- dose children based on DPT, zero-dose based on BCG, DPT, Polio and Measles, and fully -immunized children were 1.1%, 0.0% and 54.3%, respectively (S2 Table). Measles (32.9%) and BCG (2.6%) were the leading vaccines with zero-dose children overall.

### Factors associated with non-receipt of age-appropriate vaccination

Compared to children aged 6–11 months of age, children 12 months and above had a higher likelihood of missing all age-appropriate vaccines (Table 3). Similarly, compared to Kenya, all other EFGH country sites were less likely to have children missing age-appropriate vaccines with Bangladesh (adjusted Prevalence Ratio [aPR]:0.32, [95%CI: 0.28-0.36]) and Peru (aPR: 0.50; 0.45-0.56) being the least likely to have children missing vaccines. Additionally, children who came from households with ≥3 children aged <5 years (aPR:1.07;1.01-1.13), had mothers with low education (aPR:1.18; 1.12-1.24), and were wasted (Moderately: aPR: 1.06; 1.00-1.13; Severely: aPR: 1.13, 1.03-1.24) were more likely to miss all age-appropriate vaccines compared to their counterparts who did not. Moreover, children who had subsequent diarrheal episodes after the index episode were more likely to miss all age-appropriate vaccines compared to their counterparts who did not (aPR: 1.05; 1.00-1.1) (Table 3).

**Table 3:** Correlates of non-receipt of age-appropriate vaccination among children aged 6-35 months presenting with medically-attended diarrhea, EFGH *Shigella* surveillance study, June 2022 - August 2024.

Variable	Category	Bivariate		Multivariable	
		Unadjusted Prevalence Ratio [95% CI]	P-value	Adjusted Prevalence Ratio [95% CI]	P-value
Age category	6-11m	**Ref**		**Ref**	
	12-17m	**1.20 [1.13-1.27]**	**<0.001**	**1.21 [1.14-1.29]**	**<0.001**
	18-23m	**1.61 [1.52-1.71]**	**<0.001**	**1.57 [1.48-1.66]**	**<0.001**
	24-35m	**1.88 [1.78-1.98]**	**<0.001**	**1.86 [1.76-1.97]**	**<0.001**
Sex	Male	0.97 [0.93-1.01]	0.154	0.97 [0.93-1.01]	0.133
Site	Kenya	**Ref**		**Ref**	
	Bangladesh	**0.31 [0.28-0.35]**	**<0.001**	**0.32 [0.28-0.36]**	**<0.001**
	Malawi	**0.79 [0.74-0.85]**	**<0.001**	**0.78 [0.72-0.85]**	**<0.001**
	Mali	**0.58 [0.53-0.62]**	**<0.001**	**0.53 [0.47-0.58]**	**<0.001**
	Pakistan	**0.74 [0.69-0.79]**	**<0.001**	**0.65 [0.59-0.72]**	**<0.001**
	Peru	**0.51 [0.46-0.56]**	**<0.001**	**0.50 [0.45-0.56]**	**<0.001**
	The Gambia	**1.05 [1.00-1.1]**	**0.041**	**0.88 [0.81-0.95]**	**0.002**
Number of children < 5 years	1-2	**Ref**		**Ref**	
	≥ 3	**1.27 [1.22-1.32]**	**<0.001**	**1.07 [1.01-1.13]**	**0.032**
Caregiver Age Category	<20 yrs	**Ref**		**Ref**	
	20–24 yrs	1.05 [0.96-1.14]	0.264	**0.91 [0.84-0.99]**	**0.030**
	25–29 yrs	1.07 [0.98-1.16]	0.128	**0.88 [0.81-0.96]**	**0.004**
	30–34 yrs	1.08 [0.99-1.18]	0.081	0.92 [0.84-1.00]	0.059
	≥35 yrs	1.07 [0.97-1.18]	0.153	**0.86 [0.78-0.95]**	**0.002**
Wealth Quintile	Quintile 1- Least wealthy	**Ref**		**Ref**	
	Quintile 2	0.96 [0.91-1.02]	0.218	**0.93 [0.88-0.99]**	**0.019**
	Quintile 3	**0.90 [0.84-0.95]**	**0.001**	**0.92 [0.86-0.99]**	**0.018**
	Quintile 4	**0.87 [0.81-0.93]**	**<0.001**	0.98 [0.91-1.06]	0.598
	Quintile 5- Wealthiest	**0.55 [0.49-0.62]**	**<0.001**	**0.88 [0.77-0.99]**	**0.040**
Mother education^a^	Low Education	**1.35 [1.29-1.41]**	**<0.001**	**1.18 [1.12-1.24]**	**<0.001**
Father education^a^	Low Education	**1.22 [1.17-1.28]**	**<0.001**	**1.05 [1.00-1.11]**	**0.035**
Caregiver occupation^b^	No employment	**Ref**		**Ref**	
	Formal employment	0.83 [0.68-1.02]	0.081	0.91 [0.74-1.10]	0.317
	Informal employment	**1.39 [1.33-1.45]**	**<0.001**	1.00 [0.96-1.05]	0.907
Diarrhea duration in days		**0.97 [0.96-0.97]**	**<0.001**	1.00 [0.99-1.01]	0.892
Respiratory rate^c^	Normal	**Ref**		**Ref**	
	Low	**0.71 [0.64-0.79]**	**<0.001**	0.91 [0.82-1.02]	0.099
	High	**1.28 [1.19-1.38]**	**<0.001**	1.06 [0.97-1.15]	0.211
Heart rate^c^	Normal	**Ref**		**Ref**	
	Low	**0.72 [0.65-0.8]**	**<0.001**	0.90 [0.81-1.01]	0.062
	High	**1.63 [1.21-2.21]**	**0.001**	1.33 [0.89-2.01]	0.168
Dysentery	Yes	**1.09 [1.03-1.16]**	**0.004**	1.04 [0.98-1.10]	0.182
Prolonged^d^	Yes	**0.79 [0.74-0.84]**	**<0.001**	1.04 [0.95-1.13]	0.418
Persistent^e^	Yes	**0.76 [0.63-0.92]**	**0.004**	1.02 [0.81-1.27]	0.891
Dehydration^f^	None	**Ref**		**Ref**	
	Severe	**1.48 [1.33-1.66]**	**<0.001**	–	–
	Some	1.00 [0.95-1.05]	1.000	–	–
Any subsequent episodes	Yes	**0.95 [0.90-0.99]**	**0.016**	**1.05 [1.00-1.1]**	**0.045**
Modified Vesikari Score	Mild	**Ref**		**Ref**	
	Moderate	0.98 [0.92-1.04]	0.460	0.99 [0.94-1.05]	0.736
	Severe	**0.93 [0.88-0.98]**	**0.009**	1.03 [0.98-1.09]	0.269
Stunted	Yes	**1.14 [1.09-1.2]**	**<0.001**	1.00 [0.95-1.05]	0.930
Wasted	None	**Ref**		**Ref**	
	Moderate	**1.09 [1.02-1.15]**	**0.006**	**1.06 [1.00-1.13]**	**0.048**
	Severe	**1.23 [1.12-1.35]**	**<0.001**	**1.13 [1.03-1.24]**	**0.010**
Prior care-seeking	Yes	**0.68 [0.64-0.73]**	**<0.001**	–	–
seeking care in the future	Challenging	**1.19 [1.11-1.26]**	**<0.001**	–	–
Accept new vaccine	Never	**Ref**		**Ref**	
	Sometimes	1.21 [0.97-1.5]	0.094	1.10 [0.89-1.36]	0.378
	Yes	0.98 [0.8-1.21]	0.882	0.90 [0.74-1.1]	0.316
Drinking water	Improved	**Ref**		**Ref**	
	Unimproved	**1.31 [1.23-1.40]**	**<0.001**	0.99 [0.92-1.07]	0.896
Sanitation	Improved	**Ref**		**Ref**	
	Unimproved	**1.27 [1.21-1.33]**	**<0.001**	1.00 [0.95-1.05]	0.938
breastfeeding	Yes	1.08 [0.99-1.17]	0.079	1.05 [0.96-1.14]	0.265

^a^ High education- some secondary, and secondary school or greater; Low education: No education, less than primary, primary school only, and Koranic school only.

^b^ No employment- Not employed, housewife, and student; Formal employment- professional; Informal employment-business (self-employed), business (other employer), casual laborer, and farmer.

^c^ Cutoffs for heart rate and respiratory rate based on the Pediatric Advanced Life Support (PALS) guidelines.

^d^-  ≥ 7 days of diarrhea during index diarrhea episode

^e^-  ≥ 14 days of diarrhea during index diarrhea episode

^f^ Dehydration excluded from multivariable model as it’s a component of Vesikari score.

Conversely, children who came from wealthier households compared to least wealthy households (Quintile 2: (aPR: 0.93; 0.88-0.99) Quintile 3: (aPR: 0.92; 0.86-0.99) Quintile 5: (aPR: 0.88; 0.77-0.99) were less likely to miss all age-appropriate vaccinations. Compared to caregivers aged < 20 years, those who were older were less likely to have children with non-receipt of age appropriate vaccination; 20–24yrs; (aPR: 0.91; 0.84-0.99) 25–29yrs; (aPR: 0.88; 0.81-0.96); ≥ 35 yrs.; (aPR: 0.86; 0.78-0.95) (Table 3).

While we observed site-variations in the factors associated with non-receipt of age-appropriate vaccination, child’s age and mother’s education were significantly associated with non-receipt of age-appropriate vaccination across six and three sites respectively (S3 Table). Furthermore, we observed non-receipt of age appropriate vaccination to be less common in all study sites compared to Kenya but low mother’s education was positively associated with non-receipt of age-appropriate vaccinations in both infants (aPR:1.19 [1.08-1.32] and older children (aPR:1.18 [1.11-1.25] regardless of the site. Other factors observed in our study to be positively associated with non-receipt of age-appropriate vaccination included: low father’s education (aPR:1.12 [1.01-1.24], wealth quintile 4 (aPR:1.18 [1.00-1.39], dysentery (aPR:1.23 [1.09-1.40] and some dehydration (aPR:1.23 [1.10-1.38] among infants and high heart rate (aPR:1.43 [1.26-1.63] and severe wasting (aPR:1.12 [1.01-1.23] among older children (≥ 12 months) (Table 4).

**Table 4:** Age-stratified correlates of non-receipt of age-appropriate vaccination among children aged 6-35 months presenting with medically-attended diarrhea, EFGH *Shigella* surveillance study, June 2022–August 2024.

		6-11 Months				12-35 Months			
Variable	Category	Bivariate		Multivariable		Bivariate		Multivariable	
		Unadjusted Prevalence Ratio [95% CI]	P-value	Adjusted Prevalence Ratio [95% CI]	P-value	Unadjusted Prevalence Ratio [95% CI]	P-value	Adjusted Prevalence Ratio [95% CI]	P-value
Sex	Male	0.97 [0.88-1.06]	0.51	0.95 [0.87-1.04]	0.27	0.96 [0.91-1.01]	0.132	0.98 [0.93-1.03]	0.371
Site	Kenya	**Ref**		**Ref**		**Ref**		**Ref**	
	Bangladesh	**0.28 [0.23-0.34]**	**<0.001**	**0.31 [0.24-0.40]**	**<0.001**	**0.34 [0.30-0.39]**	**<0.001**	**0.35 [0.30-0.41]**	**<0.001**
	Malawi	**0.74 [0.65-0.84]**	**<0.001**	0.93 [0.79-1.09]	0.372	**0.81 [0.74-0.88]**	**<0.001**	**0.78 [0.71-0.86]**	**<0.001**
	Mali	**0.46 [0.39-0.53]**	**<0.001**	**0.49 [0.40-0.59]**	**<0.001**	**0.64 [0.59-0.71]**	**<0.001**	**0.61 [0.54-0.69]**	**<0.001**
	Pakistan	**0.73 [0.64-0.84]**	**<0.001**	**0.78 [0.66-0.93]**	**0.006**	**0.74 [0.68-0.81]**	**<0.001**	**0.69 [0.61-0.77]**	**<0.001**
	Peru	**0.22 [0.16-0.29]**	**<0.001**	**0.23 [0.17-0.32]**	**<0.001**	**0.62 [0.56-0.69]**	**<0.001**	**0.65 [0.58-0.74]**	**<0.001**
	The Gambia	**0.76 [0.67-0.85]**	**<0.001**	**0.85 [0.72-0.99]**	**0.042**	**1.14 [1.08-1.21]**	**<0.001**	1.01 [0.92-1.10]	0.910
Number of children < 5 years	1-2	**Ref**		**Ref**		**Ref**		**Ref**	
	≥ 3	**1.15 [1.05-1.27]**	**0.004**	–	–	**1.33 [1.26-1.39]**	**<0.001**	1.04 [0.97-1.11]	0.243
Caregiver Age Category	<20 yrs	**Ref**		**Ref**		**Ref**		**Ref**	
	20–24 yrs	0.99 [0.84-1.16]	0.879	–	–	1.03 [0.92-1.15]	0.620	–	–
	25–29 yrs	0.98 [0.84-1.16]	0.848	–	–	1.04 [0.93-1.16]	0.483	–	–
	30–34 yrs	1.04 [0.87-1.23]	0.673	–	–	1.06 [0.94-1.19]	0.334	–	–
	≥35 yrs	0.85 [0.69-1.04]	0.106	–	–	1.09 [0.96-1.22]	0.171	–	–
Wealth Quintile	Quintile 1- Least wealthy	**Ref**		**Ref**		**Ref**		**Ref**	
	Quintile 2	1.1 [0.94-1.28]	0.243	1.02 [0.88-1.18]	0.808	**0.91 [0.86-0.98]**	**0.007**	**0.90 [0.84-0.96]**	**0.001**
	Quintile 3	1.05 [0.90-1.23]	0.548	1.01 [0.86-1.18]	0.908	**0.85 [0.80-0.91]**	**<0.001**	**0.90 [0.84-0.96]**	**0.003**
	Quintile 4	**1.17 [1.00-1.37]**	**0.047**	**1.18 [1.00-1.39]**	**0.048**	**0.76 [0.70-0.83]**	**<0.001**	**0.89 [0.81-0.97]**	**0.009**
	Quintile 5- Wealthiest	**0.71 [0.57-0.89]**	**0.002**	1.00 [0.79-1.26]	0.995	**0.51 [0.44-0.58]**	**<0.001**	**0.83 [0.71-0.96]**	**0.015**
Mother education^a^	Low Education	**1.36 [1.24-1.5]**	**<0.001**	**1.19 [1.08-1.32]**	**0.001**	**1.37 [1.30-1.45]**	**<0.001**	**1.18 [1.11-1.25]**	**<0.001**
Father education^a^	Low Education	**1.25 [1.14-1.37]**	**<0.001**	**1.12 [1.01-1.24]**	**0.033**	**1.20 [1.14-1.26]**	**<0.001**	1.02 [0.97-1.08]	0.418
Caregiver occupation^b^	No employment	**Ref**		**Ref**		**Ref**		**Ref**	
	Formal employment	0.62 [0.37-1.05]	0.078	0.64 [0.39-1.05]	0.081	0.92 [0.74-1.14]	0.446	1.03 [0.83-1.27]	0.815
	Informal employment	**1.44 [1.31-1.59]**	**<0.001**	0.98 [0.89-1.09]	0.741	**1.35 [1.29-1.42]**	**<0.001**	1.03 [0.98-1.08]	0.292
Diarrhea duration in days		0.98 [0.97-1.00]	0.056	1 [0.99-1.02]	0.725	**0.96 [0.95-0.97]**	**<0.001**	0.99 [0.98-1.01]	0.452
Respiratory rate^c^	Normal	**Ref**		**Ref**		**Ref**		**Ref**	
	Low	**0.83 [0.72-0.97]**	**0.019**	0.94 [0.81-1.08]	0.382	0.91 [0.76-1.09]	0.323	0.93 [0.79-1.09]	0.369
	High	0.63 [0.19-2.08]	0.445	0.48 [0.16-1.42]	0.187	**1.13 [1.04-1.23]**	**0.004**	1.02 [0.94-1.11]	0.57
Heart rate^c^	Normal	**Ref**		**Ref**		**Ref**		**Ref**	
	Low	**0.80 [0.66-0.98]**	**0.029**	1.01 [0.83-1.23]	0.910	**0.71 [0.63-0.81]**	**<0.001**	**0.79 [0.69-0.89]**	**<0.001**
	High	1.68 [0.76-3.75]	0.203	1.34 [0.86-2.08]	0.193	**1.67 [1.63-1.71]**	**<0.001**	**1.43 [1.26-1.63]**	**<0.001**
Dysentery	Yes	**1.17 [1.03-1.34]**	**0.019**	**1.23 [1.09-1.40]**	**0.001**	1.03 [0.96-1.11]	0.382		
Prolonged^d^	Yes	**0.88 [0.78-0.99]**	**0.029**	1.12 [0.97-1.29]	0.130	**0.77 [0.71-0.84]**	**<0.001**	1.02 [0.91-1.14]	0.756
Persistent^e^	Yes	0.87 [0.65-1.17]	0.366	–	–	0.77 [0.59-1.01]	0.056	1.06 [0.79-1.41]	0.715
Dehydration	None	**Ref**		**Ref**		**Ref**		**Ref**	
	Severe	**1.48 [1.06-2.06]**	**0.021**	1.09 [0.80-1.5]	0.581	**1.48 [1.33-1.65]**	**<0.001**	–	–
	Some	**1.18 [1.06-1.30]**	**0.002**	**1.23 [1.1-1.38]**	**<0.001**	0.96 [0.90-1.02]	0.195	–	–
Any subsequent episodes	Yes	1.01 [0.92-1.11]	0.773	–	–	0.95 [0.90-1.00]	0.050	1.04 [0.99-1.10]	0.153
Modified Vesikari Score	Mild	**Ref**		**Ref**		**Ref**		**Ref**	
	Moderate	1.00 [0.88-1.14]	0.966	–	–	0.99 [0.92-1.06]	0.772	0.97 [0.91-1.03]	0.299
	Severe	1.02 [0.92-1.14]	0.653	–	–	**0.93 [0.87-0.99]**	**0.031**	0.97 [0.91-1.03]	0.359
Stunted	Yes	1.10 [0.97-1.25]	0.14	1.02 [0.9-1.15]	0.792	**1.06 [1.01-1.12]**	**0.028**	1.06 [1.00-1.12]	0.033
Wasted	None	**Ref**		**Ref**		**Ref**		**Ref**	
	Moderate	1.05 [0.91-1.20]	0.506	–	–	**1.10 [1.03-1.18]**	**0.005**	1.04 [0.98-1.12]	0.191
	Severe	1.15 [0.92-1.44]	0.220	–	–	**1.25 [1.13-1.38]**	**<0.001**	**1.12 [1.01-1.23]**	**0.029**
Prior care-seeking	Yes	**0.65 [0.57-0.74]**	**<0.001**	0.97 [0.85-1.11]	0.696	**0.69 [0.63-0.74]**	**<0.001**	1.00 [0.92-1.08]	0.931
seeking care in the future	Challenging	1.01 [0.86-1.19]	0.869	–	–	**1.22 [1.14-1.31]**	**<0.001**	**0.88 [0.83-0.95]**	**<0.001**
Accept new vaccine	Never	**Ref**		**Ref**		**Ref**		**Ref**	
	Sometimes	1.53 [0.94-2.5]	0.087	1.53 [0.95-2.47]	0.083	1.02 [0.81-1.28]	0.867	–	–
	Yes	1.03 [0.64-1.66]	0.887	1.13 [0.71-1.79]	0.613	0.89 [0.72-1.10]	0.290	–	–
Drinking water	Improved	**Ref**		**Ref**		**Ref**		**Ref**	
	Unimproved	**1.62 [1.43-1.83]**	**<0.001**	1.03 [0.9-1.19]	0.645	**1.24 [1.15-1.35]**	**<0.001**	0.99 [0.90-1.08]	0.776
Sanitation	Improved	**Ref**		**Ref**		**Ref**		**Ref**	
	Unimproved	**1.32 [1.19-1.47]**	**<0.001**	0.98 [0.88-1.09]	0.731	**1.29 [1.22-1.36]**	**<0.001**	1.02 [0.97-1.08]	0.427
breastfeeding	Yes	1.17 [0.94-1.46]	0.170	1.05 [0.85-1.31]	0.637	1.00 [0.91-1.11]	0.925	–	–

^a^ High education- some secondary, and secondary school or greater; Low education: No education, less than primary, primary school only, and Koranic school only.

^b^ No employment- Not employed, housewife, and student; Formal employment- professional; Informal employment-business (self-employed), business (other employer), casual laborer, and farmer.

^c^ Cutoffs for heart rate and respiratory rate based on the Pediatric Advanced Life Support (PALS) guidelines.

^d^  ≥ 7 days of diarrhea during index diarrhea episode

^e^  ≥ 14 days of diarrhea during index diarrhea episode.

## Discussion

More than half (52%) of the children enrolled in our study did not receive age-appropriate vaccination, with The Gambia reporting the highest prevalence (75.2%) and Bangladesh the lowest (22.3%). Vaccines with the highest prevalence of non-receipt of age-appropriate vaccination regardless of the site were: Polio (27%), MCV (19%) and PCV (14%) while BCG (3%) was the lowest, suggesting variations in missed opportunities for vaccination across the sites. Furthermore, predictors of non-receipt of age-appropriate vaccination included older age (24–35 months), severe wasting, caregivers taking care of three or more children <5 years in a household, low caretaker education and lower wealth index

Our findings suggest that more than half of the children enrolled in this study did not receive age-appropriate vaccination is important for several reasons. It suggests that a larger proportion of these children are inadequately protected against the intended vaccine preventable diseases, and that vaccine implementers in these settings should more than double their efforts in vaccinating children with Polio, MCV and PCV vaccines. This observation can possibly explain the persistently high infectious disease burden in these settings despite existing and routine vaccination programs. Our findings on low adherence for Polio, MCV and PCV are consistent with estimates from other recent studies showing that none of these countries may achieve the target for the third dose of Polio vaccine and measles at the regional level [21]. The reasons for delay in, or missing, the measles vaccine could include it being a single dose vaccine and that it targets children at a later age [22], consistent with observations from other resource poor settings [23]. The relatively high prevalence of delayed polio vaccination observed across several sites may reflect a combination of programmatic and measurement-related factors [24]. Polio vaccination requires multiple doses administered over time, increasing the likelihood of incomplete schedules due to missed visits or dropout. In addition, global supply constraints, particularly for IPV, and inequities in access may contribute to inconsistent availability in some settings. Differences in delivery platforms, with OPV often administered through campaigns that may not be consistently documented on vaccination cards, could further lead to underestimation of coverage. The highest adherence to BCG vaccination is consistent with previous observations [21], and could be explained by an increasing trend in hospital births that usually coincides with the vaccine’s schedule [23,25,26].

Despite the co-administration of DPT, HepB, and Hib as part of the pentavalent vaccine across all study sites, we observed discrepancies in antigen-specific coverage estimates in some countries. These differences are unlikely to reflect true variation in vaccine receipt, but rather may be attributable to heterogeneity in vaccination card documentation, data abstraction practices, and variable coding of pentavalent doses into antigen-specific fields. In settings where pentavalent vaccines are recorded as a single entry or where card completeness varies, differential misclassification of individual antigens may arise. These findings highlight the importance of standardized approaches to vaccination data capture and suggest that antigen-specific estimates derived from card data should be interpreted with caution.

Vaccination adherence declined with an increase in age, especially for vaccines scheduled later in childhood. This suggests caregivers’ domestic or socio-economic activities that may compete against taking their children for timely vaccination [27]. This finding may also be partly attributable to a cumulative probability effect, whereby older children, who are eligible for a greater number of vaccine doses, have a higher likelihood of missing at least one. This pattern may also reflect waning adherence to vaccination schedules over time and highlights the importance of strengthening follow-up and completion of multi-dose vaccine series. Worth noting from our study, is an observation that severe wasting, was associated with non-receipt of age appropriate vaccination, a finding that is consistent with literature from a systematic review by Favin et al [28].These findings may be explained in part by healthcare providers opting to defer vaccination in severely malnourished children due to safety concerns based on a perception that malnourished children may not tolerate vaccines well or may have reduced immune responses, leading to uncertainty about both safety and effectiveness as well as fear of adverse reactions [28,29]. Moreover, the association with wasting could also be explained by wasted children possibly coming from families who have less resources, less education, and overall tend to prioritize food and survival over health care attendance [30]. Additionally, three or more children under the same caregiver was also a driver of non-receipt of age-appropriate vaccination, with younger children being given priority over older children in such households, an observation which is consistent with some existing literature [5]. Our finding that children with MAD whose mothers reported lower education were more likely to have non-receipt of age-appropriate vaccination than their counterparts is in line with findings from other previous studies [31,32].

Children from relatively less wealthy or poor households were more likely to report non-receipt of age-appropriate vaccination, which is likely related to caregivers’ financial constrains which limit their free time due to competing economic engagements as a priority over taking their children for vaccination as observed in other previous studies [33,34]. Furthermore, caregivers with primary or less education were more likely to report a child with non-receipt of age-appropriate vaccination, possibly due to their lack of awareness of Expanded Programme on Immunization (EPI) schedules and associated benefits [35].

Our findings have several public health implications. First, maternal related factors are important drivers of age-appropriate vaccination in children from resource poor settings. Second, to optimize adherence to vaccine schedules, monitoring of context specific drivers of EPI schedules could improve vaccine uptake and enhance child survival strategies by targeting vulnerable children. Third, understanding factors associated with non-receipt of age-appropriate vaccination in LMICs could help inform resource allocation and strategies aimed at optimizing targeted individual vaccine uptake. Finally, should *Shigella* vaccine introduction target late infancy, then policy makers and vaccine implementers may need to consider innovative approaches to bolster its coverage given the observed EPI adherence challenges with vaccine administration in this age group [21].

Our study is subject to limitations. First, it was not powered to detect drivers of non-age-appropriate vaccination and vaccine promptness, hence our data needs to be interpreted with caution. Second, the impact of vaccine stock-outs, for instance stock-out of rotavirus vaccine in Kenya during the study period, was not evaluated in our study to help contextualize the results. Third, antenatal care data could complement and enhance understanding factors influencing receipt of age-appropriate vaccination in future studies. Fourth, inconsistent adoption of vaccines into national EPI schedules across sites, such as Bangladesh not implementing rotavirus vaccination, may have limited our ability to comprehensively capture all relevant patterns of overall non-receipt vaccination assessment. Finally, the relatively stringent inclusion and exclusion criteria inherited from the parent study limits generalizability particularly among more mobile populations and children without documented immunization histories.

## Conclusion

Our study found that the prevalence of non-receipt of age-appropriate vaccination is significantly high across the sites. Given the variation, researchers need to carry out more studies to determine the potential causes of the variation by including additional explanatory variables, such as factors connected to health care services which can be valuable for policy makers in these settings.

From the policy perspective, our study highlights the critical role of engagement with health services and caregiver education in both improving the vaccination coverage as well as improving age appropriateness of the vaccinations.

## Supporting information

S1 Checklist Inclusivity in global research. (DOCX)

S1 Table Country-specific Routine Immunization Schedules. (DOCX)

S2 Table Proportion of fully immunized and zero-dose children among children aged 6-35m presenting with medically-attended diarrhea, by EFGH sites, 2022–2024. (DOCX)

S3 Table Site-specific correlates of non-receipt of age-appropriate vaccination among children aged 6–35 months presenting with medically-attended diarrhea, EFGH *Shigella* surveillance study, June 2022–August 2024. (DOCX)
