# Supplementary material for: Frequency and correlates of non-receipt of age-appropriate vaccination among children aged 6-35 months with medically attended diarrhea: Findings from the Enterics for Global Health (EFGH) Shigella study, 2022-2024
Source: PLOS Glob Public Health. 2026 Jul 1;6(7):e0005670. doi: 10.1371/journal.pgph.0005670 (PMC13322521; doi:10.1371/journal.pgph.0005670)
Supplement: S1 Table — (DOCX) [file pgph.0005670.s002.docx]

### S1_Table: Country-specific Routine Immunization Schedules

| Vaccine | BCG | Polio (OPV/IPV) | Pentavalent  (DPT-HepB-Hib) | PCV | Rotavirus | Measles-containing vaccine |
| --- | --- | --- | --- | --- | --- | --- |
| Bangladesh | Birth | OPV: Birth, 6, 10, 14 wks;  IPV: 14 wks | 6, 10, 14 wks | 6, 10, 18 wks | 6, 10 wks | 9 months, 15 months |
| Kenya | Birth | OPV: Birth, 6, 10, 14 wks;  IPV: 14 wks | 6, 10, 14 wks | 6, 10, 14 wks | 6, 10, 14 wks | 9 months, 18 months |
| Malawi | Birth | OPV: 6, 10, 14 wks;  IPV: 14 wks | 6, 10, 14 wks | 6, 10, 14 wks | 6, 10 wks | 9 months, 15 months |
| Mali | Birth | OPV: Birth, 6, 10, 14 wks;  IPV: 14 wks | 6, 10, 14 wks | 6, 10, 14 wks | 6, 10 wks | 9 months |
| Pakistan | Birth | OPV: Birth, 6, 10, 14 wks;  IPV: 14 wks | 6, 10, 14 wks | 6, 10, 14 wks | 6, 10 wks | 9 months, 15 months |
| Peru | Birth | IPV: 2, 4, 6 months | 2, 4, 6 months | 2, 4, 12 months | 2, 4 months | 12 months, 18 months |
| The Gambia | Birth | OPV: Birth, 6, 10, 14 wks;  IPV: 4 months | 6, 10, 14 wks | 6, 10, 14 wks | 6, 10 wks | 9 months |

BCG: Bacillus Calmette-Guérin, OPV- oral polio vaccine, IPV-inactivated Polio vaccine, DPT: Diphtheria-Pertussis-Tetanus, HEPB: Hepatitis B, Hib: *Haemophilus influenzae* type b, MCV: Measles-containing vaccine (Measles+Mumps+Rubella; Measles+Rubella; Measles) and PCV: Pneumococcal conjugate vaccine
