# Supplementary material for: Frequency and correlates of non-receipt of age-appropriate vaccination among children aged 6-35 months with medically attended diarrhea: Findings from the Enterics for Global Health (EFGH) Shigella study, 2022-2024
Source: PLOS Glob Public Health. 2026 Jul 1;6(7):e0005670. doi: 10.1371/journal.pgph.0005670 (PMC13322521; doi:10.1371/journal.pgph.0005670)
Supplement: S2 Table — (DOCX) [file pgph.0005670.s003.docx]

**S2_Table**: Proportion of fully immunized and zero-dose children among children aged 6-35m presenting with medically-attended diarrhea, by EFGH sites, 2022-2024

|  | Bangladesh  % [95% CI] | Kenya  % [95% CI] | Malawi  % [95% CI] | Mali  % [95% CI] | Pakistan  % [95% CI] | Peru  % [95% CI] | The Gambia  % [95% CI] | Overall  % [95% CI] |
| --- | --- | --- | --- | --- | --- | --- | --- | --- |
| Vaccination |  |  |  |  |  |  |  |  |
| Zero DPT | 0.9[0.3-2.3] | 0.3[0.1-0.8] | 0.8[0.3-1.6] | 0.9[0.5-1.7] | 5.2[3.5-7.5] | 0.3[0-1.1] | 1.1[0.6-1.8] | 1.1[0.9-1.4] |
| Zero dose* | 0.0[0.0-0.0] | 0.0[0.0-0.0] | 0.1[0.0-0.7] | 0.1[0.0-0.6] | 0.0[0.0-0.0] | 0.0[0.0-0.0] | 0.0[0.0-0.0] | 0.0[0.0-0.1] |
| Zero BCG | 0.2[0.0-1.4] | 1.5[0.9-2.5] | 0.9[0.4-1.8] | 2.4[1.6-3.5] | 4.4[2.9-6.6] | 2.4[1.5-3.9] | 5.2[4.1-6.5] | 2.6[2.3-3.1] |
| Zero Polio** | 0.6[0.2-2.0] | 0.1[0.0-0.6] | 0.3[0.1-1] | 0.1[0.0-0.6] | 0.4[0.1-1.5] | 0.3[0.0-1.1] | 0.0[0.0-0.0] | 0.2[0.1-0.3] |
| Zero Measles*** | 35.4[31.1-39.9] | 30.6[28-33.3] | 36.7[33.5-39.9] | 34.9[32.2-37.8] | 35.8[31.7-40.1] | 46.2[42.6-49.9] | 21.8[19.7-24.1] | 32.9[31.8-34.1] |
| Fully immunized^ƛ^ | 30.2[27.0-33.5] | 71.7[68.5-74.7] | 39.2[36-42.4] | 71[67.7-74.1] | 27.4[24.6-30.4] | 51.3[47.7-55] | 88.8[86.6-90.7] | 54.3[53.1-55.6] |

DPT: Diphtheria-Pertussis-Tetanus; BCG: Bacillus Calmette-Guérin;

* Zero dose: BCG, DPT, Polio and Measles;

**OPV- oral polio vaccine, or IPV-inactivated Polio vaccine;

*** Measles-containing vaccine: (Measles+Mumps+Rubella; Measles+Rubella; Measles);

^ƛ-^Receipt of all eight doses recommended under the Expanded Programme on Immunization (EPI): one dose of BCG, three doses each of pentavalent and polio vaccines, and one dose of a measles-containing vaccine
