# Supplementary material for: Frequency and correlates of non-receipt of age-appropriate vaccination among children aged 6-35 months with medically attended diarrhea: Findings from the Enterics for Global Health (EFGH) Shigella study, 2022-2024
Source: PLOS Glob Public Health. 2026 Jul 1;6(7):e0005670. doi: 10.1371/journal.pgph.0005670 (PMC13322521; doi:10.1371/journal.pgph.0005670)
Supplement: S3 Table — (DOCX) [file pgph.0005670.s004.docx]

**Supplementary Table**

**S3_Table** : Site-specific correlates of non-receipt of age-appropriate vaccination among children aged 6-35 months presenting with medically-attended diarrhea, EFGH *Shigella* surveillance study, June 2022 - August 2024

| Variable | Category | Bangladesh | Kenya | Malawi | Mali | Pakistan | Peru | The Gambia |
| --- | --- | --- | --- | --- | --- | --- | --- | --- |
|  |  | **Adjusted Prevalence Ratio [95% CI]** | **Adjusted Prevalence Ratio [95% CI]** | **Adjusted Prevalence Ratio [95% CI]** | **Adjusted Prevalence Ratio [95% CI]** | **Adjusted Prevalence Ratio [95% CI]** | **Adjusted Prevalence Ratio [95% CI]** | **Adjusted Prevalence Ratio [95% CI]** |
| Age category | 6-11m | **Ref** | **Ref** | **Ref** | **Ref** | **Ref** | **Ref** | **Ref** |
|  | 12-17m | 1.13 [0.85-1.51] | 0.94 [0.84-1.06] | **1.17 [1.00-1.38]** | **1.45 [1.18-1.77]** | 1.03 [0.87-1.22] | 1.67 [1.14-2.44] | **1.47 [1.32-1.65]** |
|  | 18-23m | **1.59 [1.19-2.13]** | **1.34 [1.21-1.48]** | **1.32 [1.11-1.56]** | **1.42 [1.13-1.78]** | 1.16 [0.97-1.38] | 4.03 [2.88-5.62] | **1.84 [1.67-2.04]** |
|  | 24-35m | **1.44 [1.04-1.98]** | **1.59 [1.47-1.72]** | **1.29 [1.07-1.55]** | **2.61 [2.18-3.13]** | 1.41 [1.20-1.67] | 5.31 [3.87-7.28] | **2.00 [1.81-2.20]** |
| Sex | Male | 0.88 [0.71-1.09] | 0.95 [0.89-1.03] | 1.04 [0.93-1.18] | 1.07 [0.93-1.23] | 0.99 [0.88-1.11] | 1.06 [0.90-1.24] | 0.95 [0.89-1.00] |
| Number of children < 5 years | 1-2 | **Ref** | **Ref** | **Ref** | **Ref** | **Ref** | **Ref** | **Ref** |
|  | ≥ 3 | - | 0.91 [0.79-1.04] | 1.22 [0.95-1.56] | - | - | - | **1.20 [1.11-1.30]** |
| Caregiver Age Category | <20 yrs | **Ref** | **Ref** | **Ref** | **Ref** | **Ref** | **Ref** | **Ref** |
|  | 20–24 yrs | - | - | - | **0.69 [0.55-0.87]** | 0.87 [0.65-1.16] | - | - |
|  | 25–29 yrs | - | - | - | **0.75 [0.59-0.94]** | 0.93 [0.69-1.24] | - | - |
|  | 30–34 yrs | - | - | - | 0.85 [0.67-1.08] | 0.87 [0.64-1.18] | - | - |
|  | ≥35 yrs | - | - | - | **0.73 [0.57-0.95]** | 0.71 [0.51-0.99] | - | - |
| Wealth Quintile | Quintile 1- Least wealthy | **Ref** | **Ref** | **Ref** | **Ref** | **Ref** | **Ref** | **Ref** |
|  | Quintile 2 |  | **0.73 [0.63-0.85]** | 0.91 [0.75-1.10] | - | 0.94 [0.82-1.09] | 0.79 [0.65-0.97] | 1.02 [0.95-1.10] |
|  | Quintile 3 | 0.73 [0.50-1.07] | **0.72 [0.62-0.83]** | 0.85 [0.70-1.03] | - | 0.90 [0.73-1.10] | 0.73 [0.53-1.01] | **1.10 [1.01-1.19]** |
|  | Quintile 4 | 0.76 [0.52-1.11] | **0.76 [0.66-0.88]** | 0.97 [0.77-1.21] | - | 0.96 [0.77-1.20] | - | **1.21 [1.09-1.35]** |
|  | Quintile 5- Wealthiest | **0.58 [0.39-0.86]** | **0.72 [0.61-0.86]** | 0.91 [0.64-1.31] | - | 1.20 [0.85-1.70] | - | - |
| Mother education^β^ | Low Education | **1.50 [1.16-1.94]** | 1.07 [0.99-1.17] | **1.21 [1.06-1.38]** | **1.21 [1.00-1.46]** | 1.22 [1.06-1.41] | 1.04 [0.86-1.26] | 1.04 [0.96-1.12] |
| Father education^β^ | Low Education | 1.00 [0.78-1.28] | 1.04 [0.96-1.13] | 1.07 [0.91-1.25] | 1.05 [0.89-1.23] | 1.10 [0.96-1.25] |  |  |
| Caregiver occupation^¥^ | No employment | **Ref** | **Ref** | **Ref** | **Ref** | **Ref** | **Ref** | **Ref** |
|  | Formal employment | - | 0.95 [0.72-1.26] | - | 0.69 [0.39-1.24] | - | **1.55 [1.05-2.29]** | 0.57 [0.33-0.98] |
|  | Informal employment | - | 1.03 [0.95-1.11] | - | **1.60 [1.20-2.12]** | - | 0.95 [0.73-1.25] | 0.97 [0.91-1.03] |
| Diarrhea duration in days | | 1.00 [0.97-1.03] | **1.01 [1.00-1.02]** | - |  | - |  |  |
| Respiratory rate* | Normal | **Ref** | **Ref** | **Ref** | **Ref** | **Ref** | **Ref** | **Ref** |
|  | Low | - | 0.92 [0.78-1.08] | 0.71 [0.44-1.15] | 1.26 [0.95-1.66] | 0.78 [0.58-1.06] | 1.10 [0.76-1.58] | 0.88 [0.69-1.13] |
|  | High | - | 1.02 [0.84-1.25] | 1.08 [0.91-1.29] | **1.30 [1.01-1.67]** | 1.12 [0.88-1.44] | 1.06 [0.71-1.57] | 0.98 [0.87-1.11] |
| Heart rate* | Normal | **Ref** | **Ref** | **Ref** | **Ref** | **Ref** | **Ref** | **Ref** |
|  | Low | - | **1.21 [1.04-1.41]** | 1.11 [0.83-1.47] | 0.83 [0.67-1.03] | 0.82 [0.65-1.03] | - | - |
|  | High | - | 1.07 [0.64-1.78] | **1.56 [1.12-2.18]** | - | - | - | - |
| Dysentery | Yes | 1.15 [0.88-1.51] |  | 1.07 [0.88-1.29] | - | **1.26 [1.10-1.45]** | 0.96 [0.74-1.25] | 1.05 [0.98-1.13] |
| Prolonged^£^ | Yes | - | 1.01 [0.89-1.14] | - | - | - | - | - |
| Persistent^€^ | Yes | - | - | - | **3.64 [2.39-5.53]** | - | - | - |
| Dehydration | None | **Ref** | **Ref** | **Ref** | **Ref** | **Ref** | **Ref** | **Ref** |
|  | Severe | - | - | - | - | - | 1.29 [0.73-2.29] | - |
|  | Some | - | - | **1.39 [1.09-1.76]** | - | - | 1.17 [0.94-1.46] | - |
| Any subsequent episodes | Yes | - | - | **1.18 [1.04-1.33]** | - | 1.10 [0.97-1.24] | 0.84 [0.7-1.02] | - |
| Modified Vesikari Score | Mild | **Ref** | **Ref** | **Ref** | **Ref** | **Ref** | **Ref** | **Ref** |
|  | Moderate | 0.92 [0.66-1.28] | **1.10 [1.00-1.21]** | - | 1.00 [0.79-1.25] | 0.95 [0.80-1.12] | - | 0.95 [0.88-1.04] |
|  | Severe | 0.79 [0.61-1.03] | 1.08 [0.99-1.18] | - | **1.36 [1.12-1.66]** | 0.93 [0.79-1.09] | - | 1.01 [0.93-1.09] |
| Stunted | Yes | 0.94 [0.73-1.20] | 0.83 [0.75-0.92] | - | - | 1.07 [0.94-1.23] | **1.24 [1.03-1.49]** | **1.10 [1.04-1.17]** |
| Wasted | None | **Ref** | **Ref** | **Ref** | **Ref** | **Ref** | **Ref** | **Ref** |
|  | Moderate | - | **1.27 [1.09-1.49]** | - | - | **1.15 [1.01-1.32]** | 1.36 [0.99-1.86] | 0.96 [0.90-1.03] |
|  | Severe | - | 1.01 [0.66-1.54] | - | - | 1.10 [0.91-1.34] | 1.24 [0.77-2.00] | 1.09 [0.98-1.21] |
| Prior care-seeking | Yes | - | **1.13 [1.01-1.26]** | - | 0.85 [0.71-1.02] | - | - | - |
| seeking care in the future | Challenging | - | 0.62 [0.47-0.81] | - | **2.88 [2.24-3.72]** | 1.16 [0.86-1.56] |  | 0.93 [0.87-0.99] |
| Accept new vaccine | Never | **Ref** | **Ref** | **Ref** | **Ref** | **Ref** | **Ref** | **Ref** |
|  | Sometimes | - | - | 1.30 [0.96-1.76] | - | - | - | - |
|  | Yes | - | - | 0.93 [0.71-1.22] | - | - | - | **1.24 [1.03-1.49]** |
| Drinking water | Improved | **Ref** | **Ref** | **Ref** | **Ref** | **Ref** | **Ref** | **Ref** |
|  | Unimproved | - | 1.01 [0.93-1.10] | - | - | - | - | - |
| Sanitation | Improved | **Ref** | **Ref** | **Ref** | **Ref** | **Ref** | **Ref** | **Ref** |
|  | Unimproved | - | - | - | - | 0.67 [0.48-0.96] | 1.00 [0.83-1.20] | **1.24 [1.05-1.46]** |
| breastfeeding | Yes | - | - | **1.24 [1.03-1.49]** | - | 1.10 [0.91-1.33] | - | - |

^β-^ High education- some secondary, and secondary school or greater; Low education: No education, less than primary, primary school only, and Koranic school only.

^¥-^ No employment-Not employed, housewife, and student; Formal employment- professional; Informal employment-business (self-employed), business (other employer), casual laborer, and farmer.

*Cutoffs for heart rate and respiratory rate based on the Pediatric Advanced Life Support (PALS) guidelines.

^£-^≥7 days of diarrhea during index diarrhea episode

^£-^≥14 days of diarrhea during index diarrhea episode

Estimates in bold: **p<0.05**
