# Supplementary material for: Frequency and correlates of non-receipt of age-appropriate vaccination among children aged 6-35 months with medically attended diarrhea: Findings from the Enterics for Global Health (EFGH) Shigella study, 2022-2024
Source: PLOS Glob Public Health. 2026 Jul 1;6(7):e0005670. doi: 10.1371/journal.pgph.0005670 (PMC13322521; doi:10.1371/journal.pgph.0005670)
Supplement: Attachment — Submitted filename: Response to Reviewers.docx [file pgph.0005670.s006.docx]

April 21, 2026

To

Dr. Tintu Varghese, MD
Academic Editor
PLOS Global Public Health

RE: Revised Manuscript ID: PGPH-D-25-03435

Dear Dr. Tintu and Editorial Team,

Please find attached our revised manuscript (#PGPH-D-25-03435) entitled, “***Frequency and correlates of non-receipt of age-appropriate vaccination among children aged 6-35 months with medically attended diarrhea: Findings from the Enterics for Global Health (EFGH) Shigella study, 2022-2024***)” submitted for for consideration for publication as a research article for publication in the journal of PLOS Global Public Health.

We are grateful to you and the reviewers for the thoughtful and constructive feedback. We have revised the manuscript in response to all comments, which we also wish to thank you since it has improved its clarity and rigor.A detailed, point-by-point response to each comment has been uploaded separately, referencing the corresponding changes and line numbers in the revised, tracked version of the manuscript.

Thank you again for considering our resubmission. We appreciate the opportunity to improve our work and look forward to your positive evaluation.

Yours sincerely,

Caren Oreso

Kenya Medical Research Institute, Center for Global Health Research (KEMRI-CGHR)

E-mail: [oresocaren@gmail.com](mailto:oresocaren@gmail.com); Phone: +254 721 550 847

**Point-by-point response to Editor/Reviewer Comments**

We sincerely appreciate the invaluable comments and suggestions provided by the editor and the reviewers, which we believe have contributed in improving the quality of this work. Below, we present a point-by-point response (in italic) to each of the remarks, clearly highlighting the revisions made in the manuscript (with line numbers (**highlighted in bold**) referenced in the marked version) and offering clarifications where needed.

**Editorial Comments**

Journal Requirements:

**Comment 1**: Please include a complete copy of PLOS’ questionnaire on inclusivity in global research in your revised manuscript. Our policy for research in this area aims to improve transparency in the reporting of research performed outside of researchers’ own country or community. The policy applies to researchers who have travelled to a different country to conduct research, research with Indigenous populations or their lands, and research on cultural artefacts. The questionnaire can also be requested at the journal’s discretion for any other submissions, even if these conditions are not met. Please find more information on the policy and a link to download a blank copy of the questionnaire here: <https://journals.plos.org/globalpublichealth/s/best-practices-in-research-reporting>. Please upload a completed version of your questionnaire as Supporting Information when you resubmit your manuscript.

**Response 1**: *Thank you. The completed inclusivity has been completed and uploaded.*

**Comment 2**:  If the reviewer comments include a recommendation to cite specific previously published works, please review and evaluate these publications to determine whether they are relevant and should be cited. There is no requirement to cite these works unless the editor has indicated otherwise. 
**Response 2**: *Thank you for this comment. The reviewers recommend any specific references.*

**Comment 3**: Please review your reference list to ensure that it is complete and correct. If you have cited papers that have been retracted, please include the rationale for doing so in the manuscript text, or remove these references and replace them with relevant current references. Any changes to the reference list should be mentioned in the rebuttal letter that accompanies your revised manuscript. If you need to cite a retracted article, indicate the article’s retracted status in the References list and also include a citation and full reference for the retraction notice.

**Response 3:** *The reference list has been reviewed and we confirm that it is complete and correct.*

**Comment 4**: Figure Resubmissions:

While revising your submission, we strongly recommend that you use PLOS’s NAAS tool (<https://ngplosjournals.pagemajik.ai/artanalysis>) to test your figure files. NAAS can convert your figure files to the TIFF file type and meet basic requirements (such as print size, resolution), or provide you with a report on issues that do not meet our requirements and that NAAS cannot fix.

After uploading your figures to PLOS’s NAAS tool - <https://ngplosjournals.pagemajik.ai/artanalysis>, NAAS will process the files provided and display the results in the "Uploaded Files" section of the page as the processing is complete. If the uploaded figures meet our requirements (or NAAS is able to fix the files to meet our requirements), the figure will be marked as "fixed" above. If NAAS is unable to fix the files, a red "failed" label will appear above. When NAAS has confirmed that the figure files meet our requirements, please download the file via the download option, and include these NAAS processed figure files when submitting your revised manuscript.

**Response 4**: *Thank you for the comment. The Figures have been generated by NAAS and meet journal specification.*

**Decision: Minor revision.**
We invite you to revise your manuscript addressing the reviewer comments in detail. Please include a point-by-point response letter indicating how each comment has been addressed. Attention to methodological clarity, consistency in vaccine nomenclature, and additional supplementary data will enhance the manuscript’s transparency and impact.

**Reviewer 1**

**Reviewer #1: Dear Authors,**

I would like to commend you for having written this manuscript titled " Frequency and correlates of non-receipt of age-appropriate vaccination among children aged 6-35 months with medically attended diarrhea: Findings from the Enterics for Global Health (EFGH) Shigella study, 2022-2024".

I have a few comments and suggestions in the order of their appearance in the manuscript, as follows:

**Reviewer #1: Comment #1:**
1. The inclusion and exclusion criteria are rather strict, and understandably so, since this is the secondary analysis of data of recruitment for a different study - such as remaining at their current residence for at least 4 months, being excluded if presenting with non-diarrhea conditions, if a vaccination card is not available, and if more than 4 hours have passed between child presentation and study screening.
Each of these conditions would limit the generalizability of the study, despite its multicentric and multi-country nature.

**Response to Reviewer #1: Comment #1:**

*Thank you for this comment. The inclusion and exclusion criteria were largely inherited from the parent study, which was designed with specific operational and clinical objectives. While, the requirement for vaccination card availability was intended to minimize misclassification of immunization status. We acknowledge that these criteria may limit the representativeness of the study population, particularly by potentially excluding more mobile populations and children without documented vaccination records. As such, our findings may be more generalizable to care-seeking children with documented vaccination histories in similar healthcare settings, rather than to the broader pediatric population. However, the multicounty, multisite design, partially mitigates this limitation by enhancing the external validity across comparable settings. We have now clarified this limitation in the manuscript (Lines* ***449-452****).*

**Reviewer #1: Comment #2:**

2. The study has calculated age-appropriate immunization, in other words, 'timeliness' of immunization, which is extremely important; and data is hitherto sparse, if not absent.
Also, if not more important, would be a completeness of immunization, as is captured by full immunization coverage (FIC) which is the indicator of 'completeness' of immunization with routine vaccines, even if not timely. Another important parameter is the zero-dose immunization—not having received pentavalent (any DPT-containing vaccine) or any vaccine, per different definitions.
<https://pmc.ncbi.nlm.nih.gov/articles/PMC10611163/>
Notwithstanding the study having been conducted in children with medically attended diarrhoea, information on the above could make the study more comprehensive, and comparable with existing metrics. If available, it would be interesting to compare current study FIC and zero-dose data, especially >12-month data, with prevalent countrywise FIC and zero-dose data.

**Response to Reviewer #1: Comment #2:**

*Thank you for this comment. We have now presented both overall and site-specific estimates of zero-dose prevalence, based on DPT and the broader definition incorporating BCG, polio, pentavalent, and measles vaccines, as well as the proportion of fully immunized children. These results are included in Table S2 and described in the Results section (Lines* ***282-285****). We have also added a corresponding description of these definitions and analyses in the Methods section (Lines* ***204-212****).*

**Reviewer #1: Comment #3:**
3. The vaccines DPT, Hib, and Hepatitis B are administered as a combination in all countries of study conduct (Manuscript References 9-15) but have varying age-appropriate vaccination data in tables, starkly, for some countries. The authors could further explain these apparent discrepancies in discussion.

**Response to Reviewer #1: Comment #3:**

*Thank you for this comment. Overall, the most plausible explanation is that the discrepancies reflect measurement and data capture artefacts rather than true differences in vaccine uptake. These differences are unlikely to reflect true variation in vaccine receipt, but rather may be attributable to heterogeneity in vaccination card documentation, data abstraction practices, and variable coding of pentavalent doses into antigen-specific fields. The consistency across some sites and divergence in others supports the hypothesis of site-specific documentation and abstraction variability. This has been updated in the discussion section (Lines* ***383-392****).*

**Reviewer #1: Comment #4:**
4. Some countries have oral Polio, while others have inactivated and oral in their routine immunization schedules. The authors could distinguish between the two/clarify the calculation methodology for 'Polio'.
**Response to Reviewer #1: Comment #4:**

*Thank you for this comment. In the parent study, polio vaccination was captured as the total number of doses received of either oral polio vaccine (OPV) or inactivated polio vaccine (IPV), recorded collectively as “polio vaccine (OPV or IPV).”*

**Reviewer #1: Comment #5:**
5. Since some vaccines like pentavalent follow a one-month interval schedule in some countries, the authors could explain if postponement was calculated as one for each vaccine dose that was given subsequently, or as a group.

**Response to Reviewer #1: Comment #5:**

*Thank you for this clarification. In our analysis, postponement was assessed at the dose level rather than as a grouped measure. Specifically, for DPT-containing vaccines (administered as pentavalent), each scheduled dose was evaluated individually against the national immunization schedule, allowing for a window period of one month.*

**Reviewer #1: Comment #6:**
6. MMR has been mentioned in table and text, whereas most countries have Measles or MR vaccine in their schedules. The authors could clarify or correct.

**Response to Reviewer #1: Comment #6:**

*Thank you for this comment. We have replaced MMR with Measles-containing vaccine (MCV) which included the following captured separately in the parent study: Measles+Mumps+Rubella; Measles+Rubella; Measles. This has been updated in the methods (Lines* ***149-150****), results (Lines* ***278, 287, 288)*** *and Discussion (Lines* ***355).***

Thank you...

**Reviewer 2**

**Reviewer #2**: In this manuscript, the authors leverage the baseline data from the Enterics for Global Health (EFGH) study, involving seven country sites in Africa, Asia, and Latin America, to estimate the prevalence and factors associated with non-receipt of age-appropriate vaccination among children in low-and-middle income countries (LMICs). Understanding of the extent of non-receipt of age-appropriate vaccinations and the factors driving it can help design targeted interventions to strengthen immunization coverage in LMICs, where the burden of vaccine-preventable diseases is the greatest. I have a few observations that the authors may find helpful:

Major comments:

**Reviewer #2; Comment # 1:**
1. Study setting and Population: Although they are described elsewhere, a brief summary of the study setting and population, along-with the sampling plan will help the readers.

**Response to Reviewer #2: Comment #1:**

*Thank you for this comment. We have updated the section on Study setting and Population to concisely report the population and settings of the parent study (Lines* ***132-141****).*

**Reviewer #2; Comment # 2:**
2. Non-receipt of age-appropriate vaccination: It is unclear whether it is the absence of a record of having received any of the eight (seven for Bangladesh) vaccines or whether it is non-receipt of all eight vaccines. If former, it will be interesting to know the proportion of children who did not receive any vaccine, overall and by country.

**Response to Reviewer #2: Comment #2**

*Thank you for this comment. This definition referred specifically to non-receipt of all eight vaccines. We have clarified and updated the definition in the Methods section accordingly (Lines* ***159-161****). In addition, we now present estimates of both zero-dose children and fully vaccinated children aged ≥12 months in Table S2, and these results are also reported in the narrative Results section (Lines* ***282-285).***

**Reviewer #2; Comment # 3:**
3. Eligibility (inclusion and exclusion) criteria: The EFGH study included children with MAD who sought care at a designated study sentinel health facility with a vaccination card. Could this have introduced a selection bias? Also, it seems that the stated inclusion and exclusion criteria are for those participating in the EFGH study, and not specific to this analysis. Please clarify this in the manuscript.

**Response to Reviewer #2: Comment #3:**

*Thank you for this comment. We concur that the stringent eligibility criteria report may limit the generalizability of the findings especially among mobile populations and children without documented vaccination records. We have updated this in the limitations section (Lines* ***449-452****) Additionally, while most of the criteria are from the parent study having an immunization card was specific to this analysis.*

**Reviewer #2; Comment #4:**
4. Poisson regression models: Please clarify the approach used for the multivariable analysis. Was the model built using a forward or backward stepwise elimination process? Or were all variables with p-values <0.2 included and retained in the final model? How was the goodness-of-fit of the models assessed?

**Response to Reviewer #2: Comment #4:**

*We appreciate this comment. We have updated the methods section to reflect that the all variables with p-values <0.2 included and retained in the final model. Goodness of fit was evaluated was assessed using the deviance-to-degrees-of-freedom ratio. This has been updated in the methods in Lines* ***(224-229).***

**Reviewer #2; Comment #5:**
5. From the Tables 3 and 4, it appears that countries were treated as fixed effects in the regression model. Would a mixed-effects model, with countries included as random variables, be more appropriate?

**Response to Reviewer #2: Comment #5:**

*Thank you for this comment. We agree that mixed-effects models can be useful for accounting for clustering and quantifying between-site variability. However, in our analysis, we included site as a fixed effect given the relatively small number of study sites and our objective to directly estimate and interpret differences across these specific settings. Importantly, we conducted site-stratified models as a sensitivity analysis, which demonstrated some heterogeneity in associations across sites but also consistent effects for key predictors, particularly child age and caregiver education, suggesting that the main findings are robust to site-level variation. Given these considerations, the fixed-effects approach, complemented by site-specific analyses, provides a transparent and appropriate framework for our objectives. While a mixed-effects model could offer additional insight into variance partitioning, it is unlikely to materially change the primary inferences drawn from our data.*

**Reviewer #2; Comment #6:**
6. Ethical considerations: “All study participants provided written informed consent”. Was it the study participants or their parents/legally authorized representatives (LAR)? Please clarify.

**Response to Reviewer #2: Comment #6:**

*Thank you for this observation. We have updated the ethics statement to reflect that it’s the caregivers of the participants who provided written informed consent (Lines* ***238****).*

**Reviewer #2; Comment #7:**
7. Socio-economic characteristics: Please include a brief description of how the wealth quintiles were derived, adding appropriate citations if needed.

**Response to Reviewer #2: Comment #7:**

*Thank you for this comment. We have added a concise description of how the wealth quintiles were derived in the Methods section (Lines* ***166-177)*** *and included an appropriate supporting reference.*

**Reviewer #2; Comment #8:**
8. Table 2: In many countries, a large proportion of children missed the polio vaccine. Can you speculate on the possible reasons? For instance, might this be related to the multiple doses of OPV required? Additionally, the proportion of unvaccinated children appears to increase with age. Could this reflect an artifact, namely that older children, needing more vaccines overall, have a higher probability of missing at least one vaccine/dose?

**Response to Reviewer #2: Comment #8:**

*The relatively high prevalence of missed polio vaccination likely reflects the complexity of its multi-dose schedule, which increases the risk of missed doses and dropout, alongside supply constraints, particularly for IPV, and inequities in access. Additionally, reliance on campaign-based OPV delivery, which may be inconsistently documented on vaccination cards, could lead to underestimation of true coverage. This has been updated in the Discussion (Lines* ***372- 380****).*

**Minor comments:**

**Reviewer #2; Comment #1:**
1. Please consider adding a Supplementary table that outlines the country-specific immunization schedules for the age groups included in this analysis.

**Response to Reviewer #2: Comment #1:**

*Thank you for this comment. We have included Table S1 showing country-specific routine immunization schedule.*

**Reviewer #2; Comment #2:**
2. Methods, lines 153-154: “This was a secondary, cross-sectional analysis of baseline collected in the EFGH study.” Please consider revising to: “This was a secondary, cross-sectional analysis of baseline data collected in the EFGH study”.

**Response to Reviewer #2: Comment #2:**

*Thank you. This statement has been paraphrased to* ***“****This was a secondary, cross-sectional analysis of baseline data collected in the EFGH study” (Lines* ***181-182****).*

**Reviewer #2; Comment #3:**
3. Results, lines 240-244: “Similarly, compared to Kenya, all other EFGH country sites were less likely to have children missing age-appropriate vaccines with The Gambia (adjusted Prevalence Ratio [aPR]:0.91, [95%CI: 0.83-0.99]) and Malawi (aPR: 0.78; 0.72-0.85) being the least likely to have children missing vaccines.” Do you mean to state that The Gambia and Malawi were least likely to have children not missing a vaccine?

**Response to Reviewer #2: Comment #3:**

*Thank you for this comment. This was wrongly reported and has since been corrected. The countries with the least likely to have missed vaccines were Bangladesh and Peru. “all other EFGH country sites were less likely to have children missing age-appropriate vaccines with Bangladesh (adjusted Prevalence Ratio [aPR]:0.32, [95%CI: 0.28-0.36]) and Peru (aPR: 0.50; 0.45-0.56) being the least likely to have children missing vaccines”.*

**Reviewer #2; Comment #4:**
4. Figure 1: As only 31.4% of the children screened were enrolled for the study, is it possible to also include the reason(s) for screen failure, along-with the number of children in each category?

**Response to Reviewer #2: Comment #4:**

*Thank you for this comment. We have updated Fig 1 to include reasons for screening failures/ non-enrolments.*

**Reviewer #2; Comment #5:**
5. Caregiver age: Please consider categorizing the caregiver age in Tables 3, 4 and S1 for a more meaningful interpretation of the aPRs.

**Response to Reviewer #2: Comment #5:**

*Thank you for this comment. This has been updated accordingly.*

**Reviewer #2; Comment #6:**
6. Results, lines 263-265: “Similarly, children whose caregivers reported finding it challenging to seek care in the future compared to those who did not [aPR: 0.88 [0.82-0.94]] were less likely to miss all age-appropriate vaccinations.” This association seems to be driven largely by the data from Kenya (Table S1). This finding seems counterintuitive. Can you speculate on possible reasons?

**Response to Reviewer #2: Comment #6:**

*Thanks for this comment. This likely reflects selection and behavioral confounding rather than a true protective effect. Caregivers who report difficulty may paradoxically represent a subgroup that is more experienced, engaged, and persistent in navigating the health system, especially in settings where vaccination services are relatively accessible despite broader care barriers.*

**Reviewer #2; Comment #7:**
7. Results, lines 269-270: “Furthermore, we observed non-age appropriate vaccination to be less common in all study sites compared to Kenya …” Is it ‘non-age appropriate vaccination’ or ‘non-receipt of age-appropriate vaccination’?

**Response to Reviewer #2: Comment #7:**

*Thank you. This statement has been changed to “Furthermore, we observed non- receipt of age appropriate vaccination to be less common in all study sites compared to Kenya (Line* ***331****).*

**Reviewer #2; Comment #8:**
8. Results lines 266-275: Please include the aPRs and 95% CIs in the text, where applicable.

**Response to Reviewer #2: Comment #8:**

*Thank you for this observation. The aPRs and 95% CIs have been included in the text in (Lines* ***334-339).***
