# Supplementary material for: Frequency and correlates of non-receipt of age-appropriate vaccination among children aged 6-35 months with medically attended diarrhea: Findings from the Enterics for Global Health (EFGH) Shigella study, 2022-2024
Source: PLOS Glob Public Health. 2026 Jul 1;6(7):e0005670. doi: 10.1371/journal.pgph.0005670 (PMC13322521; doi:10.1371/journal.pgph.0005670)
Supplement: Peer review history

# Peer review: Decision Letter 0

**DOI:** 10.1371/journal.pgph.0005670.r001

**Authors:** Tintu Varghese

2 Mar 2026

PGPH-D-25-03435

Frequency and correlates of non-receipt of age-appropriate vaccination among children aged 6-35 months with medically attended diarrhea: Findings from the Enterics for Global Health (EFGH) Shigella study, 2022-2024

PLOS Global Public Health

Dear Dr. Caren Oreso,

Thank you for submitting your manuscript to PLOS Global Public Health. After careful consideration, we feel that it has merit but does not fully meet PLOS Global Public Health’s publication criteria as it currently stands. Therefore, we invite you to submit a revised version of the manuscript that addresses the points raised during the review process.

Please submit your revised manuscript by 28 March 2026. If you will need more time than this to complete your revisions, please reply to this message or contact the journal office at globalpubhealth@plos.org. When you're ready to submit your revision, log on to https://www.editorialmanager.com/pgph/ and select the 'Submissions Needing Revision' folder to locate your manuscript file.

Please include the following items when submitting your revised manuscript:

- A letter that responds to each point raised by the editor and reviewer(s). You should upload this letter as a separate file labeled 'Response to Reviewers'.
- A marked-up copy of your manuscript that highlights changes made to the original version. You should upload this as a separate file labeled 'Revised Manuscript with Track Changes'.
- An unmarked version of your revised paper without tracked changes. You should upload this as a separate file labeled 'Manuscript'.

Guidelines for resubmitting your figure files are available below the reviewer comments at the end of this letter.

We look forward to receiving your revised manuscript.

Kind regards,

Tintu Varghese, MD

Academic Editor

PLOS Global Public Health

Journal Requirements:

1. Please include a complete copy of PLOS’ questionnaire on inclusivity in global research in your revised manuscript. Our policy for research in this area aims to improve transparency in the reporting of research performed outside of researchers’ own country or community. The policy applies to researchers who have travelled to a different country to conduct research, research with Indigenous populations or their lands, and research on cultural artefacts. The questionnaire can also be requested at the journal’s discretion for any other submissions, even if these conditions are not met. Please find more information on the policy and a link to download a blank copy of the questionnaire here: https://journals.plos.org/globalpublichealth/s/best-practices-in-research-reporting. Please upload a completed version of your questionnaire as Supporting Information when you resubmit your manuscript.

If the reviewer comments include a recommendation to cite specific previously published works, please review and evaluate these publications to determine whether they are relevant and should be cited. There is no requirement to cite these works unless the editor has indicated otherwise.

Please review your reference list to ensure that it is complete and correct. If you have cited papers that have been retracted, please include the rationale for doing so in the manuscript text, or remove these references and replace them with relevant current references. Any changes to the reference list should be mentioned in the rebuttal letter that accompanies your revised manuscript. If you need to cite a retracted article, indicate the article’s retracted status in the References list and also include a citation and full reference for the retraction notice.

Decision: Minor revision.

We invite you to revise your manuscript addressing the reviewer comments in detail. Please include a point-by-point response letter indicating how each comment has been addressed. Attention to methodological clarity, consistency in vaccine nomenclature, and additional supplementary data will enhance the manuscript’s transparency and impact.

Reviewers' comments:

Reviewer's Responses to Questions

**Comments to the Author**

1. Does this manuscript meet PLOS Global Public Health’s publication criteria? Is the manuscript technically sound, and do the data support the conclusions? The manuscript must describe methodologically and ethically rigorous research with conclusions that are appropriately drawn based on the data presented.

Reviewer #1: Yes

Reviewer #2: Yes

**********

2. Has the statistical analysis been performed appropriately and rigorously?

Reviewer #1: Yes

Reviewer #2: Yes

**********

3. Have the authors made all data underlying the findings in their manuscript fully available (please refer to the Data Availability Statement at the start of the manuscript PDF file)?

The PLOS Data policy requires authors to make all data underlying the findings described in their manuscript fully available without restriction, with rare exception. The data should be provided as part of the manuscript or its supporting information, or deposited to a public repository. For example, in addition to summary statistics, the data points behind means, medians and variance measures should be available. If there are restrictions on publicly sharing data—e.g. participant privacy or use of data from a third party—those must be specified.

Reviewer #1: Yes

Reviewer #2: Yes

**********

4. Is the manuscript presented in an intelligible fashion and written in standard English?

PLOS Global Public Health does not copyedit accepted manuscripts, so the language in submitted articles must be clear, correct, and unambiguous. Any typographical or grammatical errors should be corrected at revision, so please note any specific errors here.

Reviewer #1: Yes

Reviewer #2: Yes

**********

5. Review Comments to the Author

Please use the space provided to explain your answers to the questions above. You may also include additional comments for the author, including concerns about dual publication, research ethics, or publication ethics. (Please upload your review as an attachment if it exceeds 20,000 characters)

Reviewer #1: Dear Authors,

I would like to commend you for having written this manuscript titled " Frequency and correlates of non-receipt of age-appropriate vaccination among children aged 6-35 months with medically attended diarrhea: Findings from the Enterics for Global Health (EFGH) Shigella study, 2022-2024".

I have a few comments and suggestions in the order of their appearance in the manuscript, as follows:

1. The inclusion and exclusion criteria are rather strict, and understandably so, since this is the secondary analysis of data of recruitment for a different study - such as remaining at their current residence for at least 4 months, being excluded if presenting with non-diarrhea conditions, if a vaccination card is not available, and if more than 4 hours have passed between child presentation and study screening.

Each of these conditions would limit the generalizability of the study, despite its multicentric and multi-country nature.

2. The study has calculated age-appropriate immunization, in other words, 'timeliness' of immunization, which is extremely important; and data is hitherto sparse, if not absent.

Also, if not more important, would be a completeness of immunization, as is captured by full immunization coverage (FIC) which is the indicator of 'completeness' of immunization with routine vaccines, even if not timely. Another important parameter is the zero-dose immunization—not having received pentavalent (any DPT-containing vaccine) or any vaccine, per different definitions.

https://pmc.ncbi.nlm.nih.gov/articles/PMC10611163/

Notwithstanding the study having been conducted in children with medically attended diarrhoea, information on the above could make the study more comprehensive, and comparable with existing metrics. If available, it would be interesting to compare current study FIC and zero-dose data, especially >12-month data, with prevalent countrywise FIC and zero-dose data.

3. The vaccines DPT, Hib, and Hepatitis B are administered as a combination in all countries of study conduct (Manuscript References 9-15) but have varying age-appropriate vaccination data in tables, starkly, for some countries. The authors could further explain these apparent discrepancies in discussion.

4. Some countries have oral Polio, while others have inactivated and oral in their routine immunization schedules. The authors could distinguish between the two/clarify the calculation methodology for 'Polio'.

5. Since some vaccines like pentavalent follow a one-month interval schedule in some countries, the authors could explain if postponement was calculated as one for each vaccine dose that was given subsequently, or as a group.

6. MMR has been mentioned in table and text, whereas most countries have Measles or MR vaccine in their schedules. The authors could clarify or correct.

Thank you...

Reviewer #2: In this manuscript, the authors leverage the baseline data from the Enterics for Global Health (EFGH) study, involving seven country sites in Africa, Asia, and Latin America, to estimate the prevalence and factors associated with non-receipt of age-appropriate vaccination among children in low-and-middle income countries (LMICs). Understanding of the extent of non-receipt of age-appropriate vaccinations and the factors driving it can help design targeted interventions to strengthen immunization coverage in LMICs, where the burden of vaccine-preventable diseases is the greatest. I have a few observations that the authors may find helpful:

Major comments:

1. Study setting and Population: Although they are described elsewhere, a brief summary of the study setting and population, along-with the sampling plan will help the readers.

2. Non-receipt of age-appropriate vaccination: It is unclear whether it is the absence of a record of having received any of the eight (seven for Bangladesh) vaccines or whether it is non-receipt of all eight vaccines. If former, it will be interesting to know the proportion of children who did not receive any vaccine, overall and by country.

3. Eligibility (inclusion and exclusion) criteria: The EFGH study included children with MAD who sought care at a designated study sentinel health facility with a vaccination card. Could this have introduced a selection bias? Also, it seems that the stated inclusion and exclusion criteria are for those participating in the EFGH study, and not specific to this analysis. Please clarify this in the manuscript.

4. Poisson regression models: Please clarify the approach used for the multivariable analysis. Was the model built using a forward or backward stepwise elimination process? Or were all variables with p-values <0.2 included and retained in the final model? How was the goodness-of-fit of the models assessed?

5. From the Tables 3 and 4, it appears that countries were treated as fixed effects in the regression model. Would a mixed-effects model, with countries included as random variables, be more appropriate?

6. Ethical considerations: “All study participants provided written informed consent”. Was it the study participants or their parents/legally authorized representatives (LAR)? Please clarify.

7. Socio-economic characteristics: Please include a brief description of how the wealth quintiles were derived, adding appropriate citations if needed.

8. Table 2: In many countries, a large proportion of children missed the polio vaccine. Can you speculate on the possible reasons? For instance, might this be related to the multiple doses of OPV required? Additionally, the proportion of unvaccinated children appears to increase with age. Could this reflect an artifact, namely that older children, needing more vaccines overall, have a higher probability of missing at least one vaccine/dose?

Minor comments:

1. Please consider adding a Supplementary table that outlines the country-specific immunization schedules for the age groups included in this analysis.

2. Methods, lines 153-154: “This was a secondary, cross-sectional analysis of baseline collected in the EFGH study.” Please consider revising to: “This was a secondary, cross-sectional analysis of baseline data collected in the EFGH study”.

3. Results, lines 240-244: “Similarly, compared to Kenya, all other EFGH country sites were less likely to have children missing age-appropriate vaccines with The Gambia (adjusted Prevalence Ratio [aPR]:0.91, [95%CI: 0.83-0.99]) and Malawi (aPR: 0.78; 0.72-0.85) being the least likely to have children missing vaccines.” Do you mean to state that The Gambia and Malawi were least likely to have children not missing a vaccine?

4. Figure 1: As only 31.4% of the children screened were enrolled for the study, is it possible to also include the reason(s) for screen failure, along-with the number of children in each category?

5. Caregiver age: Please consider categorizing the caregiver age in Tables 3, 4 and S1 for a more meaningful interpretation of the aPRs.

6. Results, lines 263-265: “Similarly, children whose caregivers reported finding it challenging to seek care in the future compared to those who did not [aPR: 0.88 [0.82-0.94]] were less likely to miss all age-appropriate vaccinations.” This association seems to be driven largely by the data from Kenya (Table S1). This finding seems counterintuitive. Can you speculate on possible reasons?

7. Results, lines 269-270: “Furthermore, we observed non-age appropriate vaccination to be less common in all study sites compared to Kenya …” Is it ‘non-age appropriate vaccination’ or ‘non-receipt of age-appropriate vaccination’?

8. Results lines 266-275: Please include the aPRs and 95% CIs in the text, where applicable.

**********

6. PLOS authors have the option to publish the peer review history of their article (what does this mean?). If published, this will include your full peer review and any attached files.

**Do you want your identity to be public for this peer review?**  If you choose “no”, your identity will remain anonymous but your review may still be made public.

For information about this choice, including consent withdrawal, please see our Privacy Policy.

Reviewer #1: No

Reviewer #2: No

**********

[NOTE: If reviewer comments were submitted as an attachment file, they will be attached to this email and accessible via the submission site. Please log into your account, locate the manuscript record, and check for the action link "View Attachments". If this link does not appear, there are no attachment files.]

Figure Resubmissions:

While revising your submission, we strongly recommend that you use PLOS’s NAAS tool (https://ngplosjournals.pagemajik.ai/artanalysis) to test your figure files. NAAS can convert your figure files to the TIFF file type and meet basic requirements (such as print size, resolution), or provide you with a report on issues that do not meet our requirements and that NAAS cannot fix.

After uploading your figures to PLOS’s NAAS tool - https://ngplosjournals.pagemajik.ai/artanalysis, NAAS will process the files provided and display the results in the "Uploaded Files" section of the page as the processing is complete. If the uploaded figures meet our requirements (or NAAS is able to fix the files to meet our requirements), the figure will be marked as "fixed" above. If NAAS is unable to fix the files, a red "failed" label will appear above. When NAAS has confirmed that the figure files meet our requirements, please download the file via the download option, and include these NAAS processed figure files when submitting your revised manuscript.

# Author comment: Author response to Decision Letter 1

**DOI:** 10.1371/journal.pgph.0005670.r002

7 May 2026

Attachment Submitted filename: Response to Reviewers.docx

# Editor report: Decision Letter 1

**DOI:** 10.1371/journal.pgph.0005670.r003

**Authors:** Tintu Varghese, Tintu Varghese

18 May 2026

Frequency and correlates of non-receipt of age-appropriate vaccination among children aged 6-35 months with medically attended diarrhea: Findings from the Enterics for Global Health (EFGH) Shigella study, 2022-2024

PGPH-D-25-03435R1

Dear Caren Oreso,

We are pleased to inform you that your manuscript 'Frequency and correlates of non-receipt of age-appropriate vaccination among children aged 6-35 months with medically attended diarrhea: Findings from the Enterics for Global Health (EFGH) Shigella study, 2022-2024' has been provisionally accepted for publication in PLOS Global Public Health.

Before your manuscript can be formally accepted you will need to complete some formatting changes, which you will receive in a follow up email. A member of our team will be in touch with a set of requests.

Please note that your manuscript will not be scheduled for publication until you have made the required changes, so a swift response is appreciated.

IMPORTANT: The editorial review process is now complete. PLOS will only permit corrections to spelling, formatting or significant scientific errors from this point onwards. Requests for major changes, or any which affect the scientific understanding of your work, will cause delays to the publication date of your manuscript.

If your institution or institutions have a press office, please notify them about your upcoming paper to help maximize its impact. If they'll be preparing press materials, please inform our press team as soon as possible -- no later than 48 hours after receiving the formal acceptance. Your manuscript will remain under strict press embargo until 2 pm Eastern Time on the date of publication. For more information, please contact globalpubhealth@plos.org.

Thank you again for supporting Open Access publishing; we are looking forward to publishing your work in PLOS Global Public Health.

Best regards,

Tintu Varghese, MD

Academic Editor

PLOS Global Public Health

***********************************************************

Dear Caren Oreso,

Thank you for submitting the revised version of your manuscript entitled, “Frequency and correlates of non-receipt of age-appropriate vaccination among children aged 6–35 months with medically attended diarrhea: Findings from the Enterics for Global Health (EFGH) Shigella study, 2022–2024.”

The revised manuscript, along with the detailed responses to reviewer comments, has been carefully evaluated. I appreciate the efforts made by the authors in addressing the reviewers’ and editors’ concerns.

I am pleased to inform you that the comments have been satisfactorily addressed, and I recommend that the manuscript be accepted for publication.

Thank you for your contribution to the journal. We look forward to publishing your work.

Best regards,

Tintu Varghese,

Academic Editor,

PLOS Global Public Health

Reviewer Comments (if any, and for reference):
